# Optimization and Enhancement of the Peroxidase-like Activity of Hemin in Aqueous Solutions of Sodium Dodecylsulfate

**DOI:** 10.1021/acsomega.3c05915

**Published:** 2023-11-03

**Authors:** Nemanja Cvjetan, Lukas D. Schuler, Takashi Ishikawa, Peter Walde

**Affiliations:** † Department of Materials, ETH-Zürich, Leopold-Ruzicka-Weg 4, 8093 Zürich, Switzerland; ‡ xirrus GmbH, Buchzelgstrasse 36, 8053 Zürich, Switzerland; § Department of Biology and Chemistry, Paul Scherrer Institute and Department of Biology, ETH-Zürich, Forschungsstrasse 111, 5232 Villigen PSI, Switzerland

## Abstract

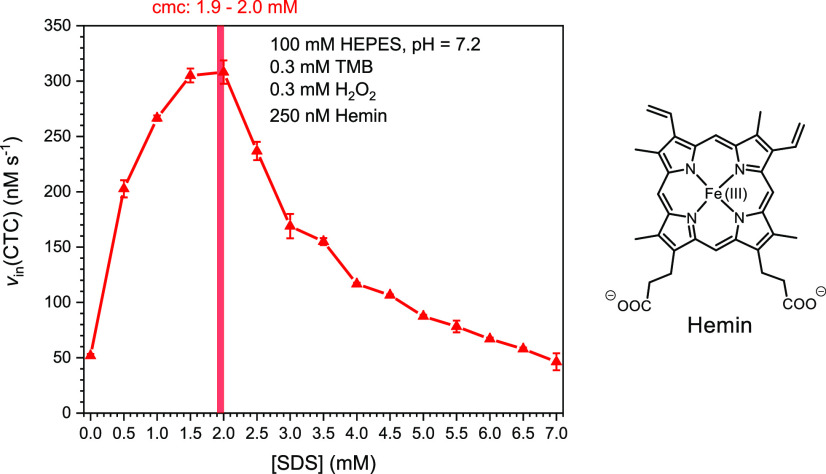

Iron porphyrins play several important roles in present-day living systems and probably already existed in very early life forms. Hemin (= ferric protoporphyrin IX = ferric heme *b*), for example, is the prosthetic group at the active site of heme peroxidases, catalyzing the oxidation of a number of different types of reducing substrates after hemin is first oxidized by hydrogen peroxide as the oxidizing substrate of the enzyme. The active site of heme peroxidases consists of a hydrophobic pocket in which hemin is embedded noncovalently and kept in place through coordination of the iron atom to a proximal histidine side chain of the protein. It is this partially hydrophobic local environment of the enzyme which determines the efficiency with which the sequential reactions of the oxidizing and reducing substrates proceed at the active site. Free hemin, which has been separated from the protein moiety of heme peroxidases, is known to aggregate in an aqueous solution and exhibits low catalytic activity. Based on previous reports on the use of surfactant micelles to solubilize free hemin in a nonaggregated state, the peroxidase-like activity of hemin in the presence of sodium dodecyl sulfate (SDS) at concentrations below and above the critical concentration for SDS micelle formation (critical micellization concentration (cmc)) was systematically investigated. In most experiments, 3,3′,5,5′-tetramethylbenzidine (TMB) was applied as a reducing substrate at pH = 7.2. The presence of SDS clearly had a positive effect on the reaction in terms of initial reaction rate and reaction yield, even at concentrations below the cmc. The highest activity correlated with the cmc value, as demonstrated for reactions at three different HEPES concentrations. The 4-(2-hydroxyethyl)-1-piperazineethanesulfonate salt (HEPES) served as a pH buffer substance and also had an accelerating effect on the reaction. At the cmc, the addition of l-histidine (l-His) resulted in a further concentration-dependent increase in the peroxidase-like activity of hemin until a maximal effect was reached at an optimal l-His concentration, probably corresponding to an ideal mono-l-His ligation to hemin. Some of the results obtained can be understood on the basis of molecular dynamics simulations, which indicated the existence of intermolecular interactions between hemin and HEPES and between hemin and SDS. Preliminary experiments with SDS/dodecanol vesicles at pH = 7.2 showed that in the presence of the vesicles, hemin exhibited similar peroxidase-like activity as in the case of SDS micelles. This supports the hypothesis that micelle- or vesicle-associated ferric or ferrous iron porphyrins may have played a role as primitive catalysts in membranous prebiotic compartment systems before cellular life emerged.

## 1 Introduction

**Figure 1:**
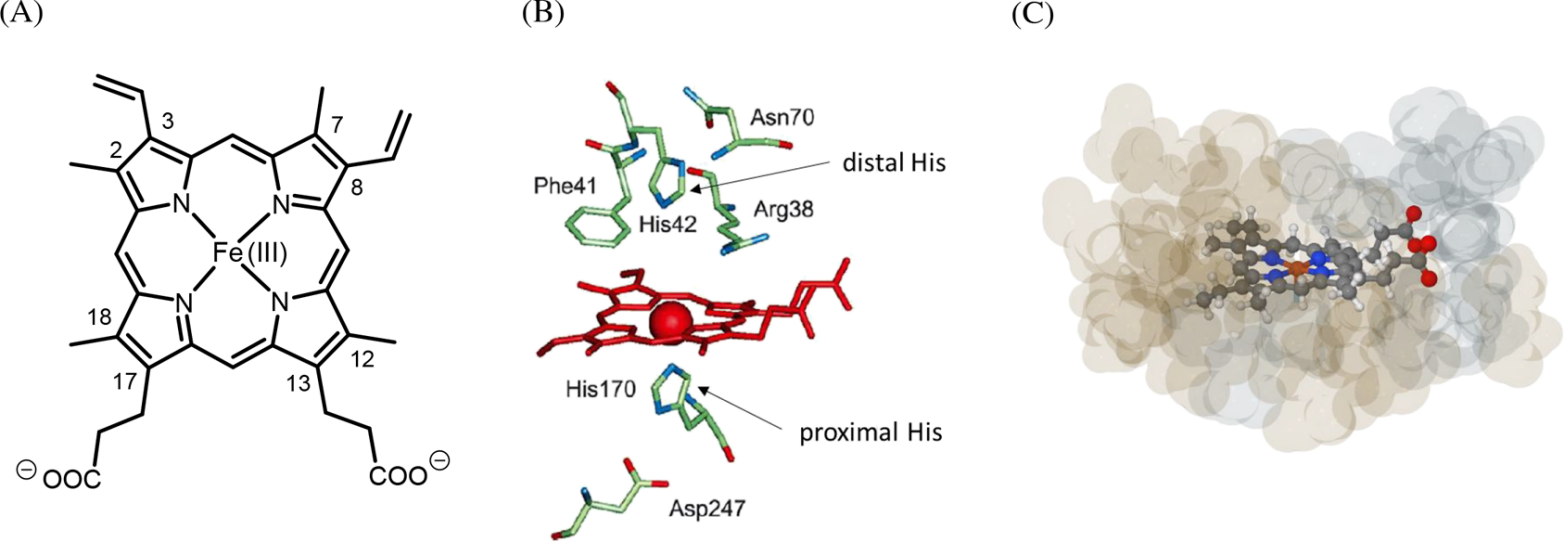
(A) Chemical structure of hemin (= (PPIX)Fe^III^ = ferric heme *b*).^1^ At the pH value used in the experiments carried out in the work presented here (pH = 7.2), the two carboxylic acids are expected to be present predominantly in deprotonated form,^4,78^ as indicated in the chemical structure. (B) Hemin as a prosthetic group at the active site of HRP, adapted from Veitch (2004).^11^ Hemin is embedded in a partially hydrophobic pocket formed by apoHRP; some of the amino acid side chains that are important for the activity of HRP are shown with proximal His170 forming a coordination bond to the ferric iron of heme *b*. (C) Illustration of the embedding of (PPIX)Fe^III^ at the active site of HRP with its hydrophobic pocket. Color code: red, O atom; blue, N atom; orange, Fe(III); brownish semitransparent, hydrophobic apoprotein surrounding; bluish semitransparent, hydrophilic apoprotein surrounding. PDB: 1HCH. (B) Reprinted from Veitch (2004)^11^ with permission from Elsevier.

Hemin, also known as ferric heme *b* (= ferric protoporphyrin IX, often abbreviated as (PPIX)Fe^III^, or (por)Fe^III^, with “por” standing for PPIX),^1^ is an organometallic compound present as a prosthetic group in various types of heme proteins,^2−4^ including small subunit monofunctional heme catalases^5−8^ and heme peroxidases^5,9−11^ (see Figure 1A). In both of these classes of enzymes, hemin is associated with the apoprotein noncovalently, kept in place mainly by hydrophobic interactions and either a Fe(III)-tyrosinate^5−8^ or a Fe(III)-histidine^5,9−11^ coordination bond. In the latter case, the free electron pair of the nonaromatic nitrogen atom of the imidazole group of a histidine residue located at the enzyme’s active site constitutes the coordination bond. For horseradish peroxidase (HRP), this histidine residue is proximal His170 (see Figure 1B). The ferric iron ion of hemin in the resting state of HRP, Fe(III), is pentacoordinated, with the four nitrogen atoms of the porphyrin ring coordinating at the four coordination sites of Fe(III) positioned in plane, and His170 coordinating at the fifth (axial) coordination site. The sixth coordination site (also axial, but opposite to the fifth coordination site) is free. This is a requirement for HRP to show efficient catalytic activity in the first step of the peroxidase cycle (binding of H_2_O_2_ and subsequent two-electron oxidation of (por)Fe^III^ to (por^+•^)Fe^IV^(O), the so-called Compound I).^4,5,9−11^ This oxidation reaction can be viewed as the removal of one electron from the iron atom, changing from Fe(III) to Fe(IV), and the removal of a second electron from PPIX, yielding a porphyrin cation radical por^+•^, as in the first step of the reactions catalyzed by monofunctional heme catalases.^7^ Overall, Compound I formation involves the heterolytic cleavage of the peroxo bond of H_2_O_2_ as an oxidizing substrate.^5,10,11^

The two vinyl groups at positions 3 and 8 (Figure 1A), together with the two methyl groups at positions 2 and 7 of the porphyrin ring, are responsible for the apolar, hydrophobic nature of one end of hemin, while the two polar propionates at positions 13 and 17 determine the hydrophilicity of the other end of hemin. With these two parts of opposite polarity and the central iron ion, hemin can be considered as an asymmetric amphiphilic compound that fits and spontaneously inserts into the accessible, partially hydrophobic pocket of the apoprotein of HRP (apoHRP) (Figure 1C). This was demonstrated in corresponding HRP reconstitution experiments in which a catalytically inactive aqueous solution of HRP from which hemin was removed and a solution of free hemin were mixed to yield reconstituted HRP with essentially the same in vitro peroxidase activity as native HRP.^12−15^ With such type of HRP reconstitution experiments it was shown, for example, (i) that the peroxidase activity—measured with *o*-dianisidine (= 3,3′-dimethoxy-4,4′-benzidine) as a reducing substrate—decreases to about one-fourth if instead of (PPIX)Fe^III^ a modified hemin is used in which the two vinyl groups are not present (so-called “deuterohemin”)^14^ or (ii) that the peroxidase activity decreases to about one-third if instead of (PPIX)Fe^III^ a modified hemin is used in which instead of the two propionic acid groups two butanoic acid groups are present (so-called “dibutyric acid hemin”).^14^ These and other similar experiments^14,15^ clearly demonstrated that the particular chemical structure of amphiphilic (PPIX)Fe^III^ matches the local environment provided by the heme binding site of HRP optimally for achieving efficient substrate conversion under physiological conditions.

Free (PPIX)Fe^III^, i.e., hemin, in aqueous solution tends to aggregate, depending on the pH, hemin concentration, and salt content.^4,16−20^ Different types of aggregates can form. Best known are catalytically inactive π–π dimers, μ-oxo dimers, μ-propionato dimers, and insoluble higher aggregates. The three kinds of dimers differ in the mode of intermolecular interactions (reflected in differences in the UV–vis absorption spectrum): aromatic π–π stacking,^4,18,19^ an oxygen atom bridging two iron atoms,^4,16,18,19,21^ and complexation between the iron atom of one (PPIX)Fe^III^ and one of the two carboxylates of a second (PPIX)Fe^III^,^4,17,21^ respectively.

The use of apoHRP-mimicking structures different from apoHRP in combination with hemin or hemin derivatives to achieve peroxidase-like activity in vitro is a challenge that various research groups have addressed in the past and are still addressing.^4^ Apart from using (i) proteinaceous scaffolds with one or more hydrophobic binding sites,^4,22−24^ (ii) sophisticated G-quadruplex RNA and DNA structures for obtaining so-called “RNAzymes” or “DNAzymes,”^25−40^ or (iii) hemin-metal–organic frameworks (MOFs) systems,^41,42^ one idea is (iv) to use aqueous micelles^25,26a,43−56^ or vesicles composed of chemically simple amphiphiles to host hemin in catalytically active state.^4^ This type of research on hemin-based, peroxidase-mimicking systems is of interest for potential biotechnological applications as cheap catalysts, for example, for oxidative oligo- or polymerization reactions.^56−59^ Furthermore, assemblies of chemically simple amphiphiles and iron porphyrins might have played a role as primitive catalysts in prebiotic protocellular structures, before the first living cells emerged from nonliving forms of matter, at the origin of life.^4^

**Figure 2:**
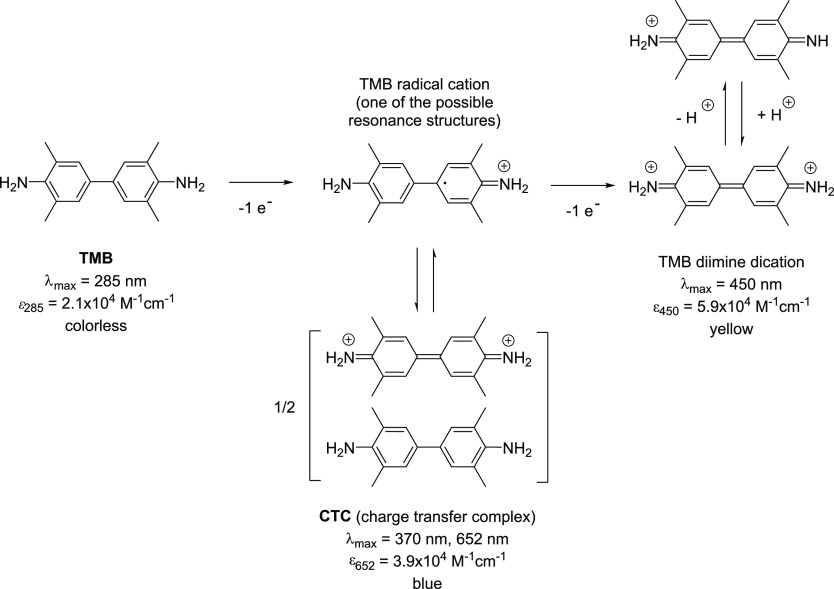
TMB and its use as a reducing substrate for detecting the peroxidase activity of HRP and of the peroxidase-like activity of hemin. The one-electron oxidation of TMB yields a blue product, known as “charge transfer complex” (CTC, blue).^61,63,66^ For the intermediate TMB radical cation, one of the possible resonance structures is shown.^66^ The two-electron oxidation product of TMB is the yellow diimine dication of TMB.^61,63,66^ Band positions of the absorption maxima are given with the corresponding molar absorption coefficient, as reported by Josephy et al.,^61^ see also Stefan et al.^29^ As shown by Fu et al.,^62^ absorption in the near-infrared region of the spectrum with λ_max_ ≈ 900 nm is another spectral feature of the blue oxidation product, which seems to support the existence of charge transfer interactions.^79^ Based on the p*K*_a_ values determined for unsubstituted benzidine^80^ and the unsubstituted benzidine dication,^81^ at pH = 7.2, the neutral form of TMB predominates, and the 2-electron oxidation product would be present predominantly as monocation.

**Figure 3:**
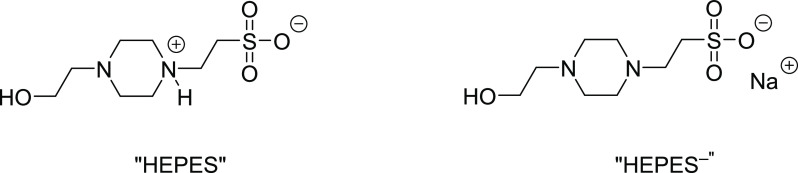
Chemical structure of HEPES in its acidic (left) and basic form (right).^75^ The abbreviations “HEPES” and “HEPES^–^“ were used in the MD simulations. The reported piperazine p*K*_a2_ value of HEPES at 25 °C is 7.6^73,74^ Therefore, at pH = 7.2, HEPES is expected to exist predominantly in its acidic form ([HEPES]:[HEPES^–^] = 2:1).

Although aqueous micelles have been used for the solubilization of hemin for investigating the spectroscopic properties in relation to the aggregation state of hemin and its axial coordination,^43,48,52^ there are not many studies dedicated to the peroxidase-like activity of hemin in the presence of micelle-forming amphiphiles.^53−57,60^ In the work presented here, the focus was on the investigation of the effect of sodium dodecylsulfate (SDS) on the peroxidase-like activity of hemin at *T* = 25 °C, by using in most of the experiments as reducing substrate 3.3′,5,5′-tetramethylbenzidine (TMB)^4,61,62^ (see Figure 2). TMB has been well-known for many years for measuring the activity of HRP^61,63−68^ or peroxidase-mimicking systems^69−72^ under acidic conditions (often pH 4–6). HEPES (= 4-(2-hydroxyethyl)-1-piperazineethanesulfonic acid)^73−75^ was applied to buffer the reaction solutions at pH = 7.2 (Figure 3), in most cases at a concentration of 100 mM. A pH value of 7.2 was arbitrarily chosen because of previous work on HRP.^76,77^ Molecular dynamics (MD) simulations were carried out to gain insight into the interaction between hemin and HEPES (as a possible axial ligand of hemin) and between hemin and SDS. In a final set of experiments, the effect of l-histidine (l-His) on the activity of hemin in the presence of HEPES and SDS was investigated, mimicking the axial ligation by proximal His170 in HRP (see above). Similar studies were carried out previously by Moosavi-Movahedi et al.^53,55^ The experimental conditions we used were, however, rather different. For some of the conditions used for the activity measurements, UV–vis absorption measurements were carried out in the Soret- and *Q*-bands regions of hemin, between λ = 250 and 800 nm,^4^ for exploring whether a correlation exists between peroxidase-like activity and characteristics of the absorption spectrum of hemin (i.e., the aggregation state of hemin).

## 2 Experimental Section

### 2.1 Materials

The following commercially available chemicals were used. Hemin (ferric protoporphyrin IX chloride), BioXtra, from porcine ≥97.0% (HPLC), product number 51280, batch numbers BCCB6735 and BCCD0941; TMB ≥ 99.0%, product number 860336, batch numbers BCBV1333 and BCCF7622; and pinacyanol chloride (= 1,1′-diethyl-2,2′-carbocyanine chloride),^82^ product number 201715, lot number 2768–90–3; ABTS^2–^(NH_4_^+^)_2_ (2,2′-azino-bis(3-ethylbenzothiazoline-6-sulfonic acid) diammonium salt), ≥98%, catalog number A1888, lot number SLCH3887; and CTAB (cetyltrimethylammonium bromide = hexadecyltrimethylammonium bromide) for molecular biology, ≈99%, product number H6269, lot number 90K0877, were from Sigma-Aldrich. Hydrogen peroxide (H_2_O_2_) 35 wt % solution in water, stabilized, catalog number 20246, lot number A0352305; HEPES for biochemistry, 99%, catalog number 17257, lot number A0233527, was from Acros Organics. SDS ≥ 99.0% (GC), BioUltra, for molecular biology, product number 71725, lot number 1361498; l-histidine (>99%), product number 53320, lot number 294377 790; and 1-dodecanol (puriss., ≥ 98.5%), product number 44100, lot number 1136831; and Triton X-100, product number 93420, lot number 1357416, were from Fluka. DCFH_2_-DA (2,7-dichlorodihydrofluorescein diacetate, ≥95%, product number 85155, lot 0523494–3, was from Cayman Chemical. SDBS (sodium dodecylbenzenesulfonate), hard type, > 95%, article number D0990, lot number FGM01, was from TCI. HRPC (horseradish peroxidase isoenzyme C), catalog number: PEO-131, grade I, lot number 8153665000, was from Toyobo Enzymes. Methanol (ACS, ISO, Reag. Ph Eur) EMSURE for analysis, product number 106009, was from Merck. DMSO (dimethyl sulfoxide) AnalaR NORMAPUR analytical reagent, ≥99.5%, catalog number 23500.297, lot number 18K084026, was from VWR Chemicals. Amplex Red (10-acetyl-3,7-dihydroxyphenoxazine), >98%, product number CDX-A0022, was from Adipogen. DCFH_2_ was prepared from DCFH_2_-DA according to the procedure reported by Ghéczy et al.^83^ In short, to 870 μL of a 10 mM sodium phosphate buffer solution (pH = 7.2), 10 μL of a 2 M NaOH solution and 100 μL of a 5 mM DCFH_2_-DA solution (prepared in DMSO and stored at 4 °C) were added. After ≈15 min, 20 μL of a 1 M HCl solution was added to readjust the pH value to 7.2. This 0.5 mM DCFH_2_ solution was then further used as obtained.

### 2.2 Preparation of Stock Solutions

Different buffer solutions of pH = 7.2 containing either 25, 50, 100, 200, or 300 mM HEPES were prepared at room temperature (RT) by dissolving HEPES in Milli-Q water and adjusting the pH value to 7.2 by addition of 2 M NaOH. Freshly prepared stock solutions of hemin (6.0 mM) and TMB (40.0 mM) in DMSO were used within ≈6 h.^67^ The 6.0 mM hemin stock solution was further diluted with DMSO to a concentration of 0.25 mM and then used as a diluted hemin stock solution. Considering the previous report of de Villiers et al.^18^ on the adsorption of hemin on the walls of plastic and quartz cuvettes, the 6.0 mM hemin stock solution was always freshly prepared and used within 1 day. H_2_O_2_ stock solutions (200 mM) were also freshly prepared with Milli-Q water and used within ≈6 h. SDS stock solutions (50 mM) were prepared in 25, 50, or 100 mM HEPES buffer solution and used within 15 days. CTAB, SDBS, and Triton X-100 stock solutions (40 mM) were prepared in a 100 mM HEPES buffer solution and used within 15 days. A 0.5 mM stock solution of pinacyanol chloride was prepared with methanol, kept at 4 °C in the dark, and used within 30 days. ABTS^2–^(NH_4_^+^)_2_ stock solutions (20 mM) were prepared in 100 mM HEPES buffer solution and used within ≈6 h. For the DCFH_2_ stock solution used (0.5 mM in 10 mM sodium phosphate buffer solution, pH = 7.2, 10% (v/v) DMSO), see Section 2.1. An Amplex Red stock solution (100 mM) was prepared in DMSO and stored at 4 °C. A l-His stock solution (60 mM) was prepared in 100 mM HEPES buffer solution pH = 7.2 and stored at RT. An HRPC stock solution was prepared by dissolving ≈4 mg HRPC in 1 mL of 100 mM sodium phosphate buffer at pH = 7.0 and stored at 4 °C. The precise HRPC concentration was determined spectrophotometrically at RT by using as molar absorption coefficient at λ = 403 nm, ε_403_(HRPC) = 102 000 M^–1^ cm^–1^,^1^ yielding 78.82 μM HRPC. This HRPC stock solution was stored at 4 °C and further diluted immediately before use, first to 0.8 μM by dissolving 10.1 μL of the 78.82 μM HRPC solution in 989.9 μL 100 mM HEPES buffer solution (pH = 7.2), followed by a further 10 times dilution with the HEPES buffer solution to yield a diluted HRPC working solution of 80 nM. This solution was used within ≈6 h.

### 2.3 Measurements of the UV–Vis Absorption Spectrum of Hemin

The UV–vis absorption spectrum of hemin under different conditions was measured with a JASCO V-670 UV–vis–NIR spectrophotometer using quartz cuvettes with a path length of 1.0 cm. For a direct comparison with the reaction carried out with hemin as a catalyst, the hemin concentration was kept constant at 250 nM, at pH = 7.2, and RT. The HEPES and SDS concentrations were varied between 25 and 300 mM (in the case of HEPES) and between 0 and 7.0 mM (in the case of SDS), respectively. The mixtures were prepared by adding appropriate volumes of the corresponding stock solutions to the cuvette. The reference cuvette contained the HEPES buffer solution. The total volume of the solutions analyzed was always 1.0 mL.

The determination of the critical concentration of SDS for micelle formation (critical micellization concentration (cmc)) in HEPES buffer solutions was carried out spectrophotometrically using the dye pinacyanol chloride,^82,84−87^ as described in the Supporting Information.

### 2.4 Determination of the Peroxidase-like Activity of Hemin Using TMB as a Reducing Substrate

The activity of hemin with TMB as reducing substrate was determined spectrophotometrically at RT with the assay developed by Josephy et al.^61^ A Specord S 600 diode array instrument from Analytic Jena and disposable polystyrene cuvettes with a path length of 1.0 cm were used. For each reaction mixture analyzed, the different components of the reaction mixture (except H_2_O_2_) were first added to the cuvette by using appropriate volumes of the stock solutions prepared in the order indicated in the legends to the figures in which the results are presented. The total volume of the reaction mixtures was always 1.0 mL and the DMSO content in the reaction mixtures was kept at 0.85 vol %, in order to reduce any effect DMSO might have on the aggregation state of hemin.^4^ After the reaction mixture was homogenized by using a micropipette, the reaction was initiated by addition of an appropriate amount of the H_2_O_2_ stock solution, immediately followed by recording at RT the UV–vis absorption spectrum at a predetermined time interval and a predetermined total reaction time (see the corresponding figure legends). The time-dependent formation of the blue one-electron oxidation product of TMB, the “CTC” obtained after a disproportionation reaction with TMB^4,61^ was followed at λ = 652 nm using ε_652_(CTC) = 39,000 M^–1^ cm^–1^.^61^ The initial rate of CTC formation, *v*_in_(CTC) (in nM s^–1^), was calculated from the slope of the initial phase of the reaction. Based on the recorded spectra for the experimental conditions used, the formation of the two-electron oxidation product of TMB, with its characteristic absorption at λ_max_ = 450 nm^61^ did not form. The reaction yield (amount of CTC formed after a chosen reaction time) was determined from *A*_652_ (without any workup of the reaction mixture) using ε_652_ (CTC) (see above).

### 2.5 Activity Measurements with ABTS^2–^, DCFH_2_, or Amplex Red as Reducing Substrates

The activity measurements with ABTS^2–^, DCFH_2_, or Amplex Red were carried out in a similar way as described for TMB in Section 2.4. The oxidation reactions were monitored by recording the absorption spectrum of the reaction mixture as a function of time and then quantifying the reaction product formation at λ = 414 or 734 nm in the case of ABTS^2–^ (using ε_414_(ABTS^•–^) = 36,000 M^–1^ cm^–1^ or ε_734_(ABTS^•–^) = 18,200 M^–1^ cm^–1^, respectively)^88^ and at λ = 571 nm in the case of Amplex Red (using ε_571_(resorufin) = 58,000 M^–1^ cm^–1^).^24,89^ For DCFH_2_, see Ghéczy et al.^83^

### 2.6 Preparation and Characterization of SDS:Dodecanol (3:7, Mol Ratio) Vesicle Dispersions and Application as Medium for the Hemin-Catalyzed Oxidation of TMB

Details about the preparation of vesicle dispersions of SDS and dodecanol in 100 mM HEPES buffer solution (pH = 7.2) based on the procedure described by Hargreaves and Deamer,^90^ and details about the polycarbonate membrane extrusion of the dispersion thus obtained using the LiposoFast device from Avestin^91^ are described in the Supporting Information. The presence of vesicles was proven by cryogenic transmission electron microscopy, as described previously by Isabettini et al.^92^ The analysis of the peroxidase-like activity of hemin in the presence of SDS:dodecanol (3:7) vesicles (pH = 7.2) was carried out in the same way as for the reactions in the presence of SDS. Details of the reaction mixture compositions are given in the figure legends.

### 2.7 MD Simulations

MD simulations were carried out using the GROMOS54A7 force field^93a^ on GROMACS^93b^ version 2019.6 running on 64bit Intel 12core CPU multithreaded. Electrostatic long-range forces were simulated using the PME method, with the Coulomb radius set equal to 1.4 nm following the GROMOS convention.

The first simulation concerned the pH = 7.2 HEPES buffer solution only, and the second one concerned hemin in HEPES buffer solution. In terms of number of species, one HEPES/hemin box contained 120 “HEPES” and 60 “HEPES^–^” species (see Figure 3 for the abbreviation used), corresponding to 100 M buffer species, 60 Na^+^ ions (as counter ions of “HEPES^–^“), 21 DMSO molecules (in the real experiment originally present in the hemin stock solution which was added to the HEPES solution, see Section 2.4), 1 hemin (deprotonated, see Figure 1A), 1 Cl^–^ ion (originally coordinating to Fe^III^), and 2 additional Na^+^ ions (as counter ions of the two PPIX carboxylates). For hemin (PPIX)Fe^III^, the charge group parameters were taken from Table S5 of the Supporting Information of the work of Zou et al.^94b^ In the case of the second HEPES/hemin simulation box, the total number of HEPES species was one fourth of the ones at 100 M HEPES, and the other composition was in both cases the same. The total volume of the simulation box was in both cases 3,000 nm^3^, the space being filled with simple point-charge (SPC) water molecules.^94b^ For this box size, simulations of 100 or 25 mM buffer species were not possible because there would be less than one molecule per box.

Note that for all MD simulations in which hemin was present, the hemin concentration in the simulation box was 533 mM (1 hemin per 3,000 nm^3^). This means that the real situation of the wet experiments (250 nM hemin) could not be represented with the MD simulations. This illustrates the limitation of the MD method for the type of system investigated in this work.

## 3 Results and Discussion

The main challenge of the work was to understand how the peroxidase-like activity of hemin in aqueous solutions of pH = 7.2 depends on the concentration of the buffer species used (HEPES), on the concentration of SDS, and on the concentration of l-His as a possible activity-promoting additive. For these three key molecules, we used a systematic experimental approach to try to find optimal (ideal) concentrations at which the peroxidase-like activity of hemin at RT toward TMB as a reducing substrate and H_2_O_2_ as an oxidizing substrate is as high as possible. In the following, results about the influence of HEPES on the peroxidase-like activity of hemin in the aqueous solution (measured with TMB as the reducing substrate) are first presented (Section 3.1), followed by a summary of data obtained about the effect of SDS on the activity of hemin in the presence of different HEPES concentrations (Section 3.2). In the same section, UV–vis absorption measurements of hemin under the conditions of the activity measurements (but in the absence of substrates) are also shown together with MD simulations as an aid for the interpretation of the kinetic data. Afterward, results about the effect of l-Hi*s* on the activity of hemin in the presence of SDS in the aqueous HEPES solution are presented (Section 3.3), followed by a comparison of the performance of the optimal micellar SDS/hemin/l-His system with the activity of HRP in the same buffer solution in the absence of SDS and l-His (Section 3.4). In Section 3.5, data about the effect of different types of micelle-forming surfactants on the activity of hemin measured with TMB as the reducing substrate are presented. In Section 3.6, results are shown for reactions run in micellar SDS solutions using reducing substrates different from TMB. Finally, preliminary results of experiments about the activity of hemin in the presence of SDS/dodecanol vesicles are summarized, again using TMB as the reducing substrate (Section 3.7).

### 3.1 Influence of HEPES on the Peroxidase-like Activity of Hemin in Aqueous Solution at pH = 7.2

In previous investigations, it was demonstrated that the activity of hemin in aqueous solution at ambient temperature not only depends on the pH value,^25^ or on the presence of hemin-complexing compounds,^4^ but also on the type and concentration of the buffer salt used.^16,55^ In experiments with hemin-binding DNA oligomers reported by Travascio et al.,^25^ the presence of HEPES or other Good’s buffers, had a positive effect on the activity of hemin (0.1 μM) at pH = 8 and 0.05% (w/v) Triton X-100, as determined with ABTS^2–^ (5 mM) as a reducing substrate and H_2_O_2_ (0.6 mM) as an oxidizing substrate.^25^ On the other hand, Moosavi-Movahedi et al.^55^ reported that for the use of sodium phosphate buffer solutions, the activity of hemin (12 μM) was found to be elevated only at low phosphate concentrations (0.2 mM) and diminished at “high” phosphate concentrations (35 mM). This was determined using guaiacol (0.22 mM) as the reducing substrate and H_2_O_2_ (1.5 mM) as the oxidizing substrate at pH = 7.4 in the presence of 16 mM SDS, with or without 3 mM imidazole.^55^

**Figure 4:**
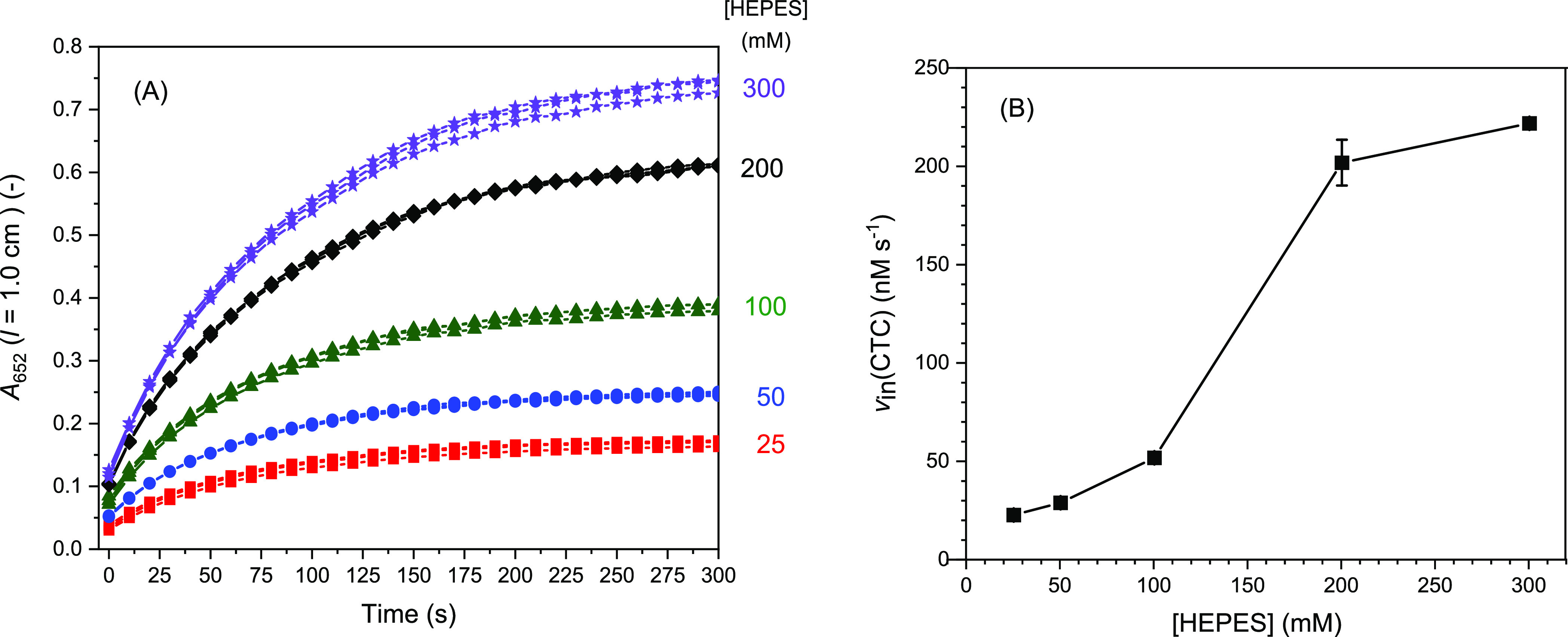
Effect of HEPES on the peroxidase-like activity of hemin measured with TMB as the reducing substrate in HEPES buffer solutions. (A) Formation of the CTC was followed by measuring *A*_652_ as a function of time for the first 300 s. (B) Initial rate of CTC formation (*v*_in_) vs HEPES concentration. Reaction conditions: [HEPES] = 25, 50, 100, 200, or 300 mM; pH = 7.2; [hemin] = 250 nM; [TMB] = 300 μM; [H_2_O_2_] = 300 μM; RT; number of measurements, *N* = 3. The conversions after 300 s were (with increasing HEPES concentration) 3, 4, 7, 10, and 12%. For the preparation of the reaction mixtures, the components were added from the corresponding stock solutions in the order the final concentrations of the components in the reaction mixtures are listed (see Section 2.4).

The first aim of our work was to further investigate how HEPES impacts the peroxidase-like activity of hemin in aqueous solution at pH = 7.2, using TMB as the reducing substrate with H_2_O_2_ as the oxidizing substrate in the absence of any additional activity-promoting compounds (no surfactants, no hemin-complexing molecules). A pH value of 7.2 was chosen to later allow a direct comparison with previous activity measurements of HRP.^76,77^ After an initial screening with different substrates, TMB was chosen because its oxidation could be measured spectrophotometrically with high sensitivity.^95,96^ The initial concentrations of TMB and H_2_O_2_ were kept constant at 300 μM. The HEPES concentration was varied between 25 and 300 mM; see Section 2.4 for experimental details. The activity of hemin (250 nM) was evaluated by recording the entire absorption spectrum of the reaction mixture between λ = 320 and 900 nm during the first 5 min after starting the reaction (see Figure S1 in the Supporting Information). The time evolution of the absorption spectrum clearly showed the appearance of absorption bands at λ = 370, 652, and ≈900 nm, typical for the formation of the CTC (TMB diimine cation + TMB, Figure 2),^61,62,79^ caused by the one-electron oxidation of TMB to the TMB radical, followed by a disproportionation reaction involving a second TMB molecule (see Josephy et al.^61^ and Cvjetan and Walde).^4^ There was no indication of the formation of a band centered at λ = 450 nm (which is typical for the formation of uncomplexed diamine dication, Figure 2).^4,61^ The spectral changes were analyzed in terms of the time-dependent increase of the absorbance at λ = 652 nm (Figure 4A), from which the initial rate of CTC formation, abbreviated as *v*_in_(CTC), was calculated, taking into account ε_652_(CTC) = 39,000 M^–1^ cm^–1^^61^ (see Section 2.4). A plot of *v*_in_ vs molar concentration of HEPES is shown in Figure 4B. The data of Figure 4 clearly show that the reaction yield after 5 min and the initial rate of CTC formation increased with increasing HEPES concentration. Therefore, there is no doubt that the presence of HEPES has a positive effect on the reaction (Figure 4A). There is, however, a leveling-off of the reaction after about 300 s for all HEPES concentrations used. The dependence of *v*_in_(CTC) on the HEPES concentration is sigmoidal (Figure 4B). The reason for this behavior is not clear. It seems to be a consequence of the various possible interactions between the different species present in the reaction mixture (in addition to a large number of water molecules): HEPES, hemin, TMB, and CTC (see later in Section 5).

**Figure 5:**
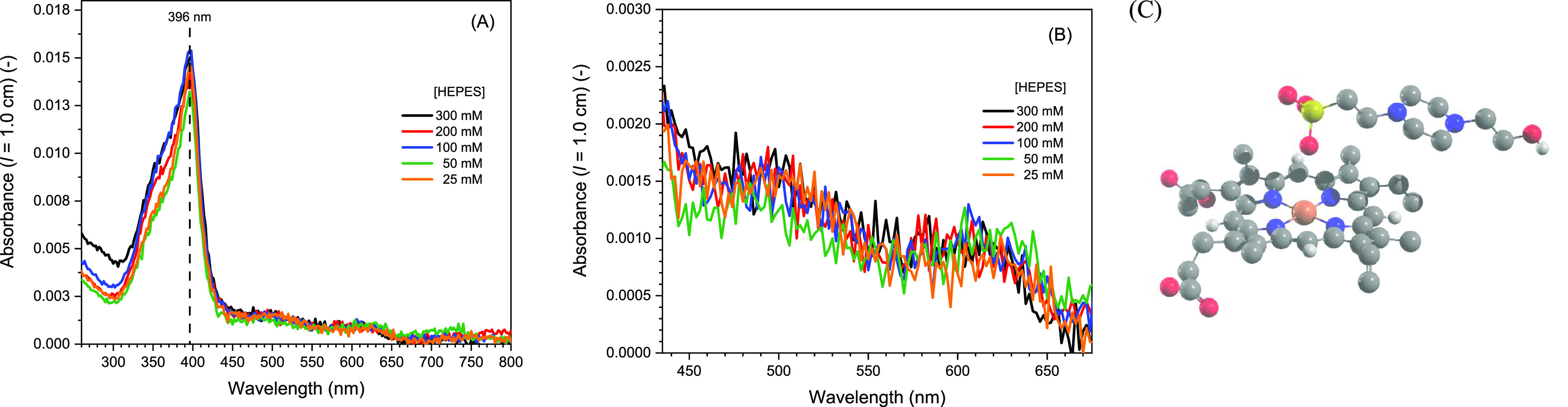
(A) UV–vis absorption spectrum of hemin in either 25, 50, 100, 200, or 300 mM HEPES buffer solution at [hemin] = 250 nM, pH = 7.2, RT. The calculated molar absorption coefficient of hemin in 100 mM HEPES solution (measured *A*_396_ = 0.015) is ε_396_ ≈ 60,000 M^–1^ cm^–1^. This value is between ε_393_ = 79,000 M^–1^ cm^–1^ determined by Kannan et al.^98^ for hemin in aqueous solution at pH = 7.2 (ionic strength 0.154 M) and ε_393_ = 45,000 M^–1^ cm^–1^ reported by de Villiers et al.^18^ for the hemin π–π dimer at pH = 7.2 (ionic strength 0.154 M). (B) Zoom-in of the Q-bands region. (C) Illustration of the coordination of one HEPES molecule to hemin, as obtained by MD simulations, see also the Supporting Information. The MD simulations with 100 and 25 mM HEPES at pH 7.2 revealed that if HEPES gets into the vicinity of hemin, at least one deprotonated HEPES (“HEPES^–^”) preferably binds to the iron center of hemin through the sulfonate. Some clustering of up to four HEPES molecules around hemin was observed, but its structure varied. Color code: white, H atom; gray, C atom; red, O atom; blue, N atom, yellow, S atom; orange, Fe(III).

There are at least two possible explanations to qualitatively rationalize the observed increase of *v*_in_(CTC) with an increase in HEPES concentration. It could be that with increasing HEPES concentration (i) the percentage of monomeric hemin increased, or (ii) HEPES acted as an axial ligand, and with this it increased the oxidizability of the ferric iron, Fe(III). To probe whether the relative amount of monomeric hemin increases with increasing HEPES concentration, UV–vis absorption measurements of hemin were carried out (see Figures 5A,B). The Soret band intensity and position are well-known to be sensitive to changes in the aggregation state of hemin.^4,19,97^ As can be seen from Figure 5A, there was no significant change in the hemin absorption spectrum in the Soret band region as a function of the HEPES concentration between 25 and 300 mM (no marked change in band position and intensity). Furthermore, there was also no change in the more sensitive Q-bands region of the spectrum between 435 and 675 nm (Figure 5B). This implies that the amount of monomeric hemin stayed approximately the same independent of the HEPES concentration; i.e., the observed increase of *v*_in_(CTC) with increasing HEPES concentration cannot be due to a significant HEPES-induced change in hemin’s aggregation state.

MD simulations were carried out to hopefully get some hint about possible hemin–HEPES interactions. In the first step, simulations of only HEPES (25 or 100 M) in water at pH = 7.2 were carried out. The results obtained show that the HEPES molecules form small dynamic “clusters,” assemblies of several HEPES molecules, the number and size of the clusters being higher at 100 M than at 25 M (see Figure S2 in the Supporting Information). In a second step, simulations were made in 25 and 100 M HEPES solution of pH = 7.2 containing one hemin molecule per simulation box (533 mM). From the two simulations of HEPES in the presence of hemin at pH = 7.2, the following was observed: (i) one water molecule coordinates in the axial position through the O atom and (ii) the sulfonate O atoms and the hydroxyl O atom of “HEPES” and “HEPES^–^“ on average are closer to Fe(III) than the N atom of “HEPES” or “HEPES^–^” (see Figure 5C and Movie S1 in the Supporting Information). Overall, it could be that interactions between HEPES and hemin are responsible for the observed increase of *v*_in_(CTC) with increasing HEPES concentration (Figure 4B). A direct quantitative comparison of the activity data and the UV-vis absorption measurements of hemin with the MD simulation was, however, not possible due to the large difference between the hemin concentration in the wet measurements (250 nM) and the situation in the MD simulation (533 mM) (see Section 2.7).

Concerning the leveling-off of the amount of formed CTC after 5 min of reaction (Figure 4A), three possible explanations are (i) the amount of H_2_O_2_ added at the beginning of the reaction was limiting; (ii) H_2_O_2_ caused hemin inactivation (degradation), as was reported to be the case for G-quadruplex/hemin systems^28,40^; and (iii) the CTC interacted with hemin thereby preventing the reaction from proceeding further (product inhibition). Possibility (i) could be excluded by one of the experiments reported in Figure S3 in the Supporting Information: For the reaction run in 100 mM HEPES (pH = 7.2), addition of a new portion of H_2_O_2_ after 5 min of reaction did not increase the CTC yield, excluding H_2_O_2_ as a limiting factor. A second experiment supported possibility (iii) and excluded possibility (ii), also shown in Figure S3 in the Supporting Information: The amount of CTC formed continued to increase if, after 5 min of reaction, SDS (2.0 mM final concentration) was added, indicating that hemin was not degraded but continued to be catalytically active. The reaction proceeded with continued formation of the one-electron oxidation product. This implies that SDS most likely weakened intermolecular contacts between hemin and the CTC (possibly disrupting π–π stacking interactions), thereby freeing hemin, which then became catalytically active again. The presence of strong hemin–CTC interactions would explain why the yellow two-electron oxidation product with λ_max_ = 450 nm (Figure 2) did not form. Moreover, in a recently published study, the catalytic activity of HRP was measured with TMB as a reducing substrate in the presence of SDS, and it was found that SDS interacts with the CTC, preventing the formation of the two-electron oxidation product even in the case of HRP.^68^ The absence of the formation of a two-electron oxidation product is what we observed in all reactions run with hemin as a catalyst in the presence of SDS with H_2_O_2_ as the oxidant (see Section 3.2).

To conclude this part of the work, from a practical point of view for possible applications of any type of hemin-based peroxidase-mimicking systems, it is of utmost importance to analyze the reactions not only in terms of initial rates of substrate oxidation but also in terms of substrate conversion (reaction yield). This was also recognized before in the work of Solomon et al.^99^ by using hemin complexed to peptide amphiphiles at pH = 7.0 and TMB as a reducing substrate. As shown in Figure 4A, for “free” hemin in the HEPES buffer solution, the substrate conversion was always low. As shown in the next section, the situation changed completely for the reactions run in the presence of SDS.

### 3.2 Influence of SDS on the Peroxidase-like Activity of Hemin in HEPES Solution of pH = 7.2

To the best of our knowledge, in all previous reports on the activity of hemin in aqueous solution in the presence of SDS, the SDS concentration used was always above the cmc,^53,55,100^ the SDS micelles considered to serve as hosts for keeping hemin in monomeric,^43,48,52^ catalytically active state (see also Section 1).

**Figure 6:**
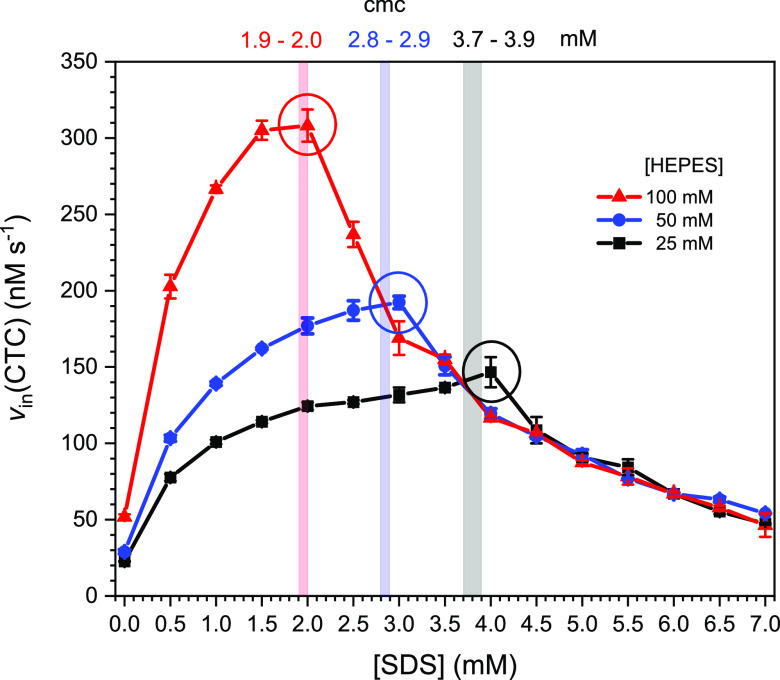
Dependence of the peroxidase-like activity of hemin in HEPES buffer solution on the concentrations of SDS and HEPES, measured with TMB as the reducing substrate. The initial rate of CTC formation, *v*_in_(CTC), is plotted against the SDS concentration. Reaction conditions: [HEPES] = 25, 50, or 100 mM; pH = 7.2; [SDS] = 0.0–7.0 mM (in 0.5 mM steps); [hemin] = 250 nM, [TMB] = 300 μM, [H_2_O_2_] = 300 μM; RT, *N* = 3. The highest *v*_in_(CTC) values for each HEPES concentration are encircled. The vertical bars indicate the determined cmc values; see Figures S4B–S6B in the Supporting Information.

In our work, we have explored the effect of SDS on the peroxidase-like activity of hemin in HEPES buffer solution for SDS concentrations below as well as above the cmc, up to 7.0 mM, for [HEPES] = 25, 50, or 100 mM, at pH = 7.2 and RT, using TMB as reducing substrate at 300 μM and [H_2_O_2_] = 300 μM. The results are shown in Figure 6 as plot of *v*_in_(CTC) vs SDS concentration. The cmc of SDS was determined for each HEPES concentration using the dye pinacyanol chloride,^82,84−86^ see Figures S4–S6 in the Supporting Information. The data are listed in Figure 6. The higher the HEPES concentration, the lower the cmc: 3.7–3.9, 2.8–2.9, and 1.9–2.0 mM for 25, 50, and 100 mM HEPES, respectively. As a reference, the cmc of SDS was also determined in 100 mM sodium phosphate buffer solution (pH = 7.2, 25 °C): 0.6–0.8 mM (see Figure S7 in the Supporting Information).

For each HEPES concentration, *v*_in_(CTC) first increased with increasing SDS concentration until the maximal value was reached after which *v*_in_(CTC) was decreased again. Before discussing this behavior, four specific observations are worth emphasizing. (i) The SDS concentration above which the hemin activity started to decrease correlates with the cmc; (ii) at the cmc, *v*_in_(CTC) was 6–7 times higher than without SDS: 6.3 times for 25 mM HEPES, 6.8 times for 50 mM HEPES, and 5.9 times for 100 mM HEPES, respectively; (iii) above 4 mM SDS, *v*_in_(CTC) was independent of the HEPES concentration, steadily decreasing with increasing SDS concentration up to at least 7.0 mM; and (iv) there was no leveling-off of the CTC formation with reaction time if SDS was present (see Figures S8 and S9 in the Supporting Information). This is consistent with the data shown in Figure S3 (increase in *A*_652_ after SDS addition for 300 s). In Figure S9, it is also shown that in the absence of hemin or H_2_O_2_, but under otherwise identical reaction conditions, the oxidation of TMB was insignificant. For data about the effect of SDS on the stability of aqueous hemin solutions, see Figure S10.

**Figure 7:**
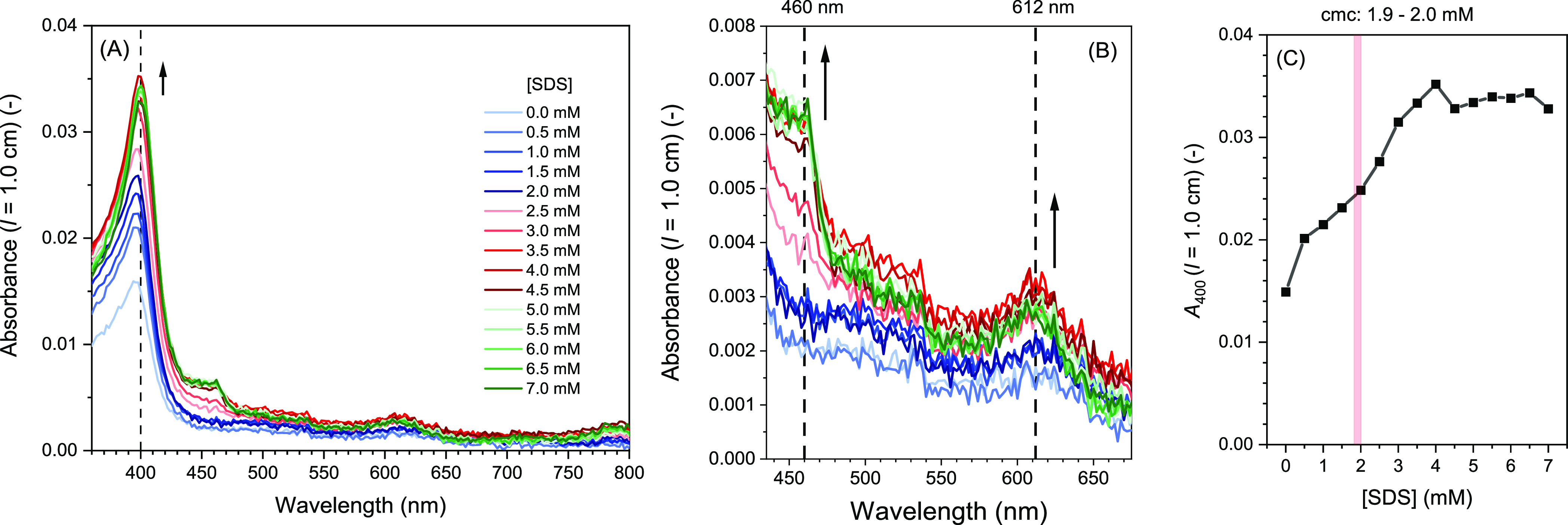
Effect of SDS on the UV–vis absorption spectrum of hemin (250 nM) in a 100 mM HEPES buffer solution (pH = 7.2) at RT. [SDS] = 0.0–7.0 mM (in 0.5 mM steps). The order for adding the corresponding stock solutions for preparing the samples was: 1. HEPES; 2. SDS; 3. Hemin. (A) Entire spectrum recorded between λ = 350 and 800 nm. (B) Zoom-in of the *Q*-bands region. For the labels, see (A). (C) *A*_400_ vs SDS concentration. The vertical bar indicates the determined cmc (see Figure S4B in the Supporting Information).

In a first attempt toward an understanding of the behavior shown in Figure 6, the UV–vis absorption spectrum of hemin in 100 mM HEPES buffer solution (pH = 7.2) in the presence of increasing amounts of SDS was measured. The hemin concentration was kept the same as in the case of the activity measurements (250 nM) and the SDS concentration was varied from 0 to 7.0 mM (see Figure 7).

With increasing SDS concentration, the band intensities increased up to [SDS] ≈ 4 mM, above which the spectrum remained unchanged (see Figure 7C in which *A*_400_ is plotted against the SDS concentration). For [hemin] = 250 nM, *A*_400_ (*l* = 1.0 cm) yields ε_400_ = 120,000 M^–1^ cm^–1^, which compares with the reported spectrum and molar absorption coefficient of monomeric, pentacoordinated hemin, (PPIX)Fe^III^(OH), determined at pH = 8.3 (0.2 M sodium phosphate buffer solution) in the presence of 345 mM SDS (10 wt %) ([hemin] = 20–60 μM), as reported by Boffi et al.^52^ The presence of a Q-band at λ_max_ ≈ 610 nm (Figure 7A) is also a characteristic feature of the hemin spectrum reported by Boffi et al.^52^ (see also Cvjetan and Walde).^4^

Overall, the absorption spectra shown in Figure 7A and B indicate that, under the conditions used, hemin is present in a monomeric, nonaggregated state to at least some degree in the presence of SDS—both below and above the cmc. Below the cmc, the amount of monomeric hemin steadily increased with increasing SDS concentration up to the cmc and above, leveling-off at about 4 mM (Figure 7C). Therefore, the decrease in peroxidase-like activity above the cmc (>2.0 mM, Figure 6), cannot be ascribed to the formation of hemin aggregates from which one would expect a reduction in activity.^4,101,102^ Therefore, there must be another reason for the hemin activity decrease above the cmc.

**Figure 8:**
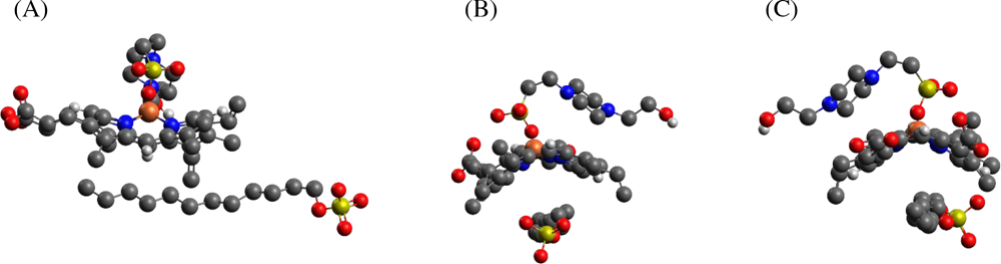
Illustration of the interaction between hemin (center), HEPES (top), and SDS (bottom). Three views of the same snapshot after 150 ns are shown: side view of the SDS molecule (A), view onto the polar headgroup of SDS (B), and view onto the tail of SDS (C). For the color code, see Figure 5B. The persistence of the interactions between hemin and SDS and between hemin and HEPES is shown in Movie S1 (Supporting Information). See also Movie S2 (Supporting Information).

The possible interaction of SDS with monomeric hemin was investigated by MD simulations (2.0 and 50 M SDS, 533 mM hemin; see Figure 8, Movies S1 and S2 in the Supporting Information).

The MD simulations not only show the formation of small SDS aggregates at high SDS concentration (possibly the formation of premicellar assemblies)^103^ but also indicate that an SDS molecule can adsorb on one side of hemin. This occurs with contacts between the hydrophobic dodecyl chain and PPIX, and with a positioning of the anionic sulfate headgroup in the opposite direction of the two propionate residues of hemin, see Figure 8. This type of interaction is conceptually like the alignment of two hemin molecules with respect to each other in a π–π dimer.^4,18,19^ One of the two molecules in the simulation box relatively quickly interacted with an SDS molecule (after about 89 ns), and the bound SDS molecule remained bound throughout the simulation period (up to 190 ns) (see Movie S1 in the Supporting Information). A further characteristic feature of the binding of SDS to hemin is the bending of hemin toward the SDS molecule (see Figure 8). It could be that such type of SDS–hemin interactions results in a decrease in the amount of dimeric hemin as the SDS concentration is increased, and therefore lead to an increase of the peroxidase-like activity of (monomeric) hemin, until a maximal value is reached.

At SDS concentrations above the cmc, but still below the concentration at which the micelles undergo a phase transition to form a hexagonal phase (≈ 35 wt % = 121 mM),^104^ the concentration of micelles increases with increasing SDS concentration. This means that the micelles hosting hemin more and more sequester the hydrophobic substrate (TMB), thereby causing a decrease in its local concentration and explaining the observed lower rate of CTC formation. Such changes in the local substrate concentration as the concentration of micelles increases were discussed in a number of previous studies in which a decrease in reaction rates in micellar systems with increasing surfactant concentration were observed.^105−110^ As a conclusion from this consideration, the aggregation state of hemin in systems of aggregate-forming amphiphiles may not correlate in a simple way with the catalytic activity of hemin. Noncovalent interactions between the reducing substrate used for measuring the activity and the aggregates formed by the surfactants must also be considered. Conditions for obtaining hemin in a monomeric state are necessary but insufficient for obtaining an efficiently functioning peroxidase-mimicking system.

Concerning the fact that at [SDS] > cmc, for example, at [SDS] = 6 mM, *v*_in_(CTC) became independent of the HEPES concentration may indicate that hemin is no longer able to bind HEPES in the presence of SDS micelles, eliminating the beneficial effect of HEPES at [SDS] > cmc. An alternative explanation could be that the sequestering effect of SDS micelles on TMB above the cmc dominates over the positive effect of HEPES. Future studies should shed some light on this.

### 3.3 Influence of l-His on the Peroxidase-like Activity of Hemin in 100 mM HEPES Solution of pH = 7.2 Containing SDS

**Figure 9:**
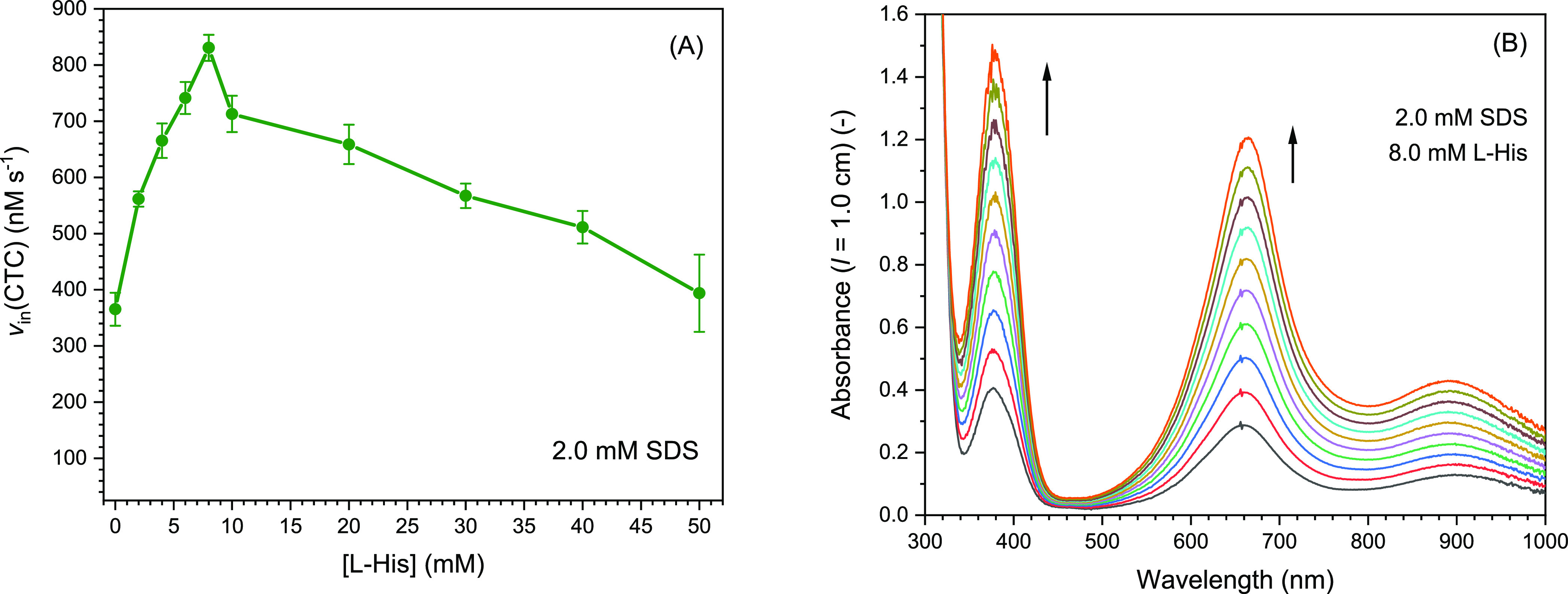
Dependence of the peroxidase-like activity of hemin in 100 mM HEPES buffer solution (pH = 7.2) containing 2.0 mM SDS on the concentration of l-His, measured with TMB as the reducing substrate. (A) Initial rate of CTC formation is plotted against the l-His concentration. Reaction conditions: [HEPES] = 100 mM; pH = 7.2; [SDS] = 2.0 mM; [hemin] = 250 nM; [l-His] = 0.0, 2.0, 4.0, 6.0, 8.0, 10, 20, 30, 40, or 50 mM; [TMB] = 0.3 mM; [H_2_O_2_] = 0.3 mM; RT; *N* = 3. (B) Reaction progress for the conditions mentioned above with [l-His] = 8.0 mM. The UV–vis absorption spectrum of the reaction mixture was measured every 3 s for a total of 30 s.

In the next series of measurements, the possible effect of l-histidine (l-His) on the peroxidase-like activity of hemin in 100 mM HEPES buffer solution of pH = 7.2 was investigated by using TMB as a reducing substrate. The SDS concentration used was 2.0 mM, based on the results shown in Figure 6. l-His was chosen as potential activity-promoting additive due to (i) the presence of proximal His170 as an important axial hemin ligand for efficient substrate conversions in the case of HRP (see Section 1 and Figure 1B) and (ii) the known axial coordination of histidine-containing peptides or imidazole to free hemin (see, for example, Casella et al.,^111^ Uno et al.,^112^ Casella et al.,^113^ Boffi et al.,^52^ and Moosavi-Mohavedi et al.^53^). For the optimal SDS concentration of 2.0 mM at [HEPES] = 100 mM (pH = 7.2) and [hemin] = 250 nM, [TMB] = [H_2_O_2_] = 0.3 mM (see Figure 6), the determined dependence of *v*_in_(CTC) on the concentration of l-His (up to 50 mM) is shown in Figure 9 (see also the *A*_652_ vs. time data in Figure S11 in the Supporting Information). From Figure 9, it is clear that *v*_in_(CTC) first steadily increased with increasing l-His concentration up to 8.0 mM and then decreased again, reaching at 50 mM a level similar to the one measured without added l-His.

**Figure 10:**
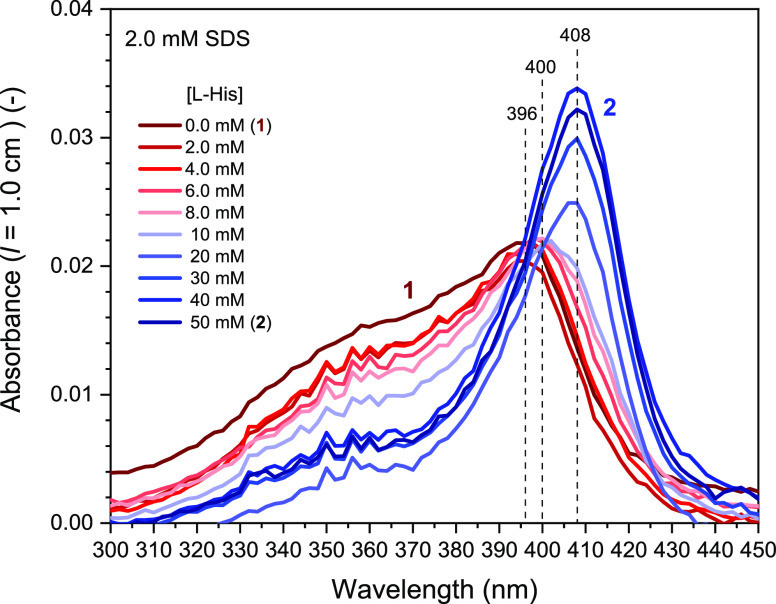
Effect of l-His on the Soret band region of the UV–vis absorption spectrum of hemin (250 nM) in a 100 mM HEPES buffer solution (pH = 7.2) containing 2.0 mM SDS. [l-His] = 0.0, 2.0, 4.0, 8.0, 10, 20, 30, 40, and 50 mM. The order of addition of the appropriate stock solutions to prepare the samples was: 1. HEPES; 2. SDS; 3. Hemin; 4. l-His.

The increase in *v*_in_(CTC) is thought to be a consequence of the interaction of l-His with hemin, most likely via the expected coordination of the nonaromatic nitrogen atom of the imidazoyl group to Fe(III) of hemin (see Section 1). The p*K*_a_ value of the protonated form of the imidazole group of free histidine at 25 °C is p*K*a ≈ 6.3.^114^ Therefore, at pH = 7.2, most of the histidine side chains are likely to be present in basic form, prone to forming the expected coordination bond, yielding at low l-His concentration “mono-l-His-ligated” hemin (possibly replacing bound HEPES). The imidazoyl p*K*_a_ value of l-His may, however, vary depending on the local environment in which l-His is present in the SDS solution, in analogy to the influence of the microenvironment of proteins has on p*K*_a_ of internal histidine side chains of the proteins (variations of ± ≈1 p*K*_a_ unit).^115^ In any case, for the chosen concentrations of HEPES, hemin, TMB, and H_2_O_2_ at pH = 7.2, the presence of 8.0 mM l-His was found to have the largest effect on the initial rate of CTC formation, *v*_in_(CTC) being about 2.2 times higher than that without l-His. The observed drop in the reaction rate for [l-His] = 10–50 mM may be due to the formation of “bis-l-His-ligated” hemin. In bis-l-His ligated hemin, both of hemin’s axial coordination positions are occupied by l-His, thereby hindering hydrogen peroxide from approaching Fe^III^ of hemin for starting the reaction (formation of Compound I, see Section 1). The existence of mono- and bis-l-His-ligated hemin species, depending on the concentration of l-His in the presence of 2.0 mM SDS, is supported by the UV–vis absorption measurements, as shown in Figure 10. A bathochromic (red) shift of the Soret peak of hemin was observed upon increasing the concentration of l-His. In the absence of l-His, hemin’s Soret band is positioned at λ_max_ ≈ 396 nm, while with increasing l-His concentration, the band position shifted to λ_max_ ≈ 408 nm (clearly seen for [l-His] = 50.0 mM), at [l-His] = 8.0 mM λ_max_ being 403 nm. A similar change in the UV–vis absorption spectrum was reported by Casella et al.^111^ for a deuterohemin-undecapeptide derivative to which imidazole was added in methanol as solvent (shift of λ_max_ of the Soret band from 388 to 400 nm).

Control experiments carried out in the presence of different amounts of l-His but in the absence of SDS showed a significantly lower rate of CTC formation than if SDS was present (Figure S12 in the Supporting Information). Additionally, the CTC band intensity at λ_max_ = 652 nm, *A*_652_, started to level off at a very low CTC yield (only 7–10% of the maximally possible amount of CTC formed after 90 s), different from what was observed for the reactions run in the presence of SDS (Figure S11 in the Supporting Information).

Overall, the investigations with l-His as potentially activity-promoting additive demonstrated that the addition of l-His can indeed have a positive effect on the peroxidase-like activity of hemin in aqueous HEPES buffer solution of pH = 7.2 in the presence of SDS. This is consistent with the conclusions drawn previously by Moosavi-Movahedi et al.^53^ for experimental conditions that were very different from those we used. Paying attention to the experimental conditions is important when aiming for high reaction rates and yields. In our work, the experiments with TMB as a reducing substrate showed that the following conditions reproducibly result in relatively high *v*_in_(CTC) values and high conversions: [HEPES] = 100 mM; pH = 7.2; [SDS] = 2.0 mM; [hemin] = 250 nM; [l-His] = 8.0 mM; [TMB] = 0.3 mM, [H_2_O_2_] = 0.3 mM; RT. The initial rate of CTC formation was ≈850 nM s^–1^ with a TMB conversion after 60 min of about 87% (see Figure S13 in the Supporting Information). In the next step, we tried to compare this peroxidase-like activity of the SDS/hemin/l-His system to the peroxidase activity of HRPC.

### 3.4 Comparison of the Micellar SDS/Hemin/l-His System with HRPC in Terms of TMB Oxidation with H_2_O_2_ as an Oxidizing Substrate

**Figure 11:**
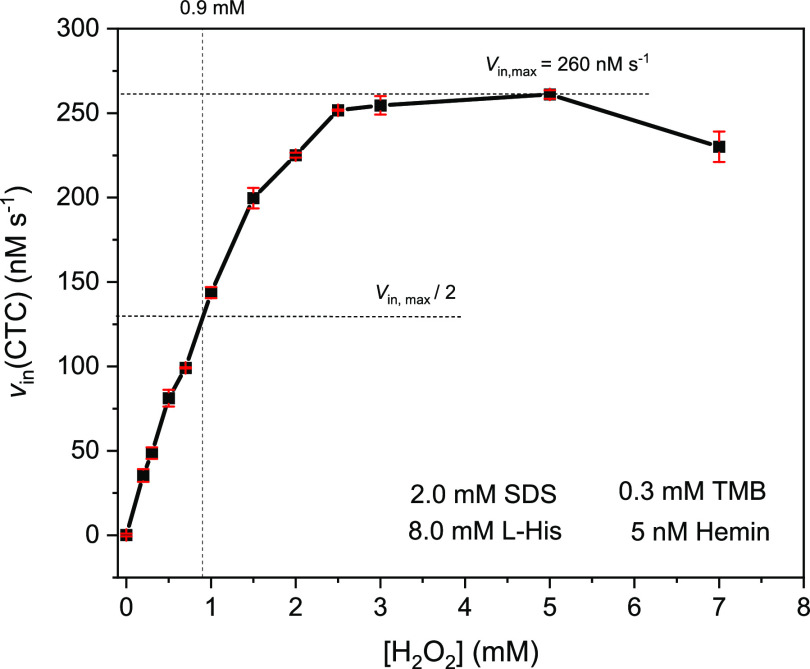
Dependence of the initial rate of CTC formation from TMB (0.3 mM) on the hydrogen peroxide concentration for the SDS/hemin/l-His system in 100 mM HEPES buffer solution (pH = 7.2) containing 5 nM hemin, 2.0 mM SDS, and 8.0 mM l-His. The reaction was run at RT with [H_2_O_2_] = 0.0, 0.2, 0.3, 0.5, 0.7, 1.0, 1.5, 2.0, 2.5, 3.0, 5.0, and 7.0 mM; *N* = 3. The experiments were performed using the following order of addition of the corresponding stock solutions: 1. HEPES; 2. SDS; 3. Hemin; 4. l-His; 5. TMB; 6. H_2_O_2_.

Some of the characteristics of the peroxidase-like activity of the SDS/hemin/l-His system toward TMB as a reducing substrate in 100 mM HEPES buffer solution (pH = 7.2) were investigated. In a first series of measurements, *v*_in_(CTC) for the elaborated optimal conditions in terms of concentrations of SDS (2.0 mM), l-His (8.0 mM), and TMB (0.3 mM) was determined for different concentrations of H_2_O_2_ as an oxidizing substrate (up to 7.0 mM) (see Figure 11). Due to the fast reaction rates for [H_2_O_2_] > 0.3 mM, the concentration of hemin had to be reduced from 250 to 5 nM for practical reasons.

Figure 11 shows that *v*_in_(CTC) first increased with an increasing H_2_O_2_ concentration until a maximum of *v*_in,max_(CTC) = 260 nM s^–1^ was reached at 2.5–5.0 mM. At 7.0 mM H_2_O_2_, *v*_in_(CTC) decreased to ≈230 nM s^–1^. Although the data shown in Figure 11 do not obey Michaelis–Menten kinetics,^116,117^ it is possible to calculate an apparent catalytic constant (apparent turnover number), “*k*_cat,app_(H_2_O_2_)” = *v*_in,max_(CTC)/[hemin] (= 260 nM s^–1^/5 nM = 52 s^–1^), which resembles *k*_cat_ in the case of enzymes obeying Michaelis–Menten kinetics (see Table 1). An apparent Michaelis constant, “*K*_M,app_(H_2_O_2_),” for the SDS/hemin/l-His system was determined as the concentration of H_2_O_2_ at which *v*_in_(CTC) reached half the value of *v*_in,max_(CTC), yielding 900 μM (see Table 1).

**Table 1:** Comparison of the Apparent Michaelis-Menten Constants Determined for the Optimal SDS/Hemin/l-His System with the Michaelis-Menten Constants for the Enzyme HRPC, Determined in 100 mM HEPES Buffer Solution (pH = 7.2)

	H_2_O_2_ (oxidizing substrate)			TMB (reducing substrate)		
	*k*_cat_ (s^–1^)	*K*_M_ (μM)	*k*_cat_/*K*_M_ (M^–1^·s^–1^)	*k*_cat_ (s^–1^)	*K*_M_ (μM)	*k*_cat_/*K*_M_ (M^–1^·s^–1^)
SDS/hemin/l-His^a^	52	900	5.8 × 10^4^	39 ± 3	253 ± 37	1.5 × 10^5^
HRPC^b^	95	56	1.7 × 10^6^	not determined^c^		

a Reaction conditions: [HEPES] = 100 mM; pH = 7.2; [SDS] = 2.0 mM; [hemin] = 5 nM; [l-His] = 8.0 mM; RT (see Figures 11, 12, and Figure S14 in the Supporting Information). For H_2_O_2,_ [TMB] = 0.3 mM and [H_2_O_2_] varied between 0.0 and 3.0 mM. For TMB, [TMB] was varied between 0 and 0.4 mM and [H_2_O_2_] = 1.0 mM.

b Reaction conditions: [HEPES] = 100 mM; pH = 7.2; [HRPC] = 5 nM; RT (see Figure S15 in the Supporting Information). For H_2_O_2,_ [TMB] = 0.3 mM and [H_2_O_2_] varied between 0 and 0.3 mM.

c The determination was not possible due to the formation of the two-electron oxidation product (see Figure 2). In previous investigations under slightly different experimental conditions, *K*_M_(TMB) was found to be 100 ± 10 μM (in 50 mM sodium phosphate buffer at pH = 7.4)^118^ and 247 μM (in 50 mM citrate-phosphate buffer at pH = 5.5),^119^ respectively. This suggests that the concentration of TMB used (0.3 mM) for the determination of *K*_M_(H_2_O_2_) and *k*_cat_(H_2_O_2_) for HRPC was probably sufficient to ensure pseudo-first-order kinetics.

In further experiments, variation of the concentration of TMB showed again saturation kinetics in terms of *v*_in_(CTC), whereby a maximal rate of CTC formation could not be reached experimentally (see Figure 12 and Figure S14 in the Supporting Information). Nevertheless, this optimal SDS hemin/l-His system was then treated as enzyme-like entity, and *v*_in,max_(CTC) was determined by fitting the experimental data obtained for [hemin] = 5 nM and [H_2_O_2_] = 1.0 mM with the Michaelis–Menten equation,^116,117^ yielding “*k*_cat,app_(TMB)” = 39 ± 3 s^–1^ and “*K*_M,app_(TMB)” = 253 ± 37 μM (see Figure 12, Figure S14 in the Supporting Information, and Table 1).

**Figure 12:**
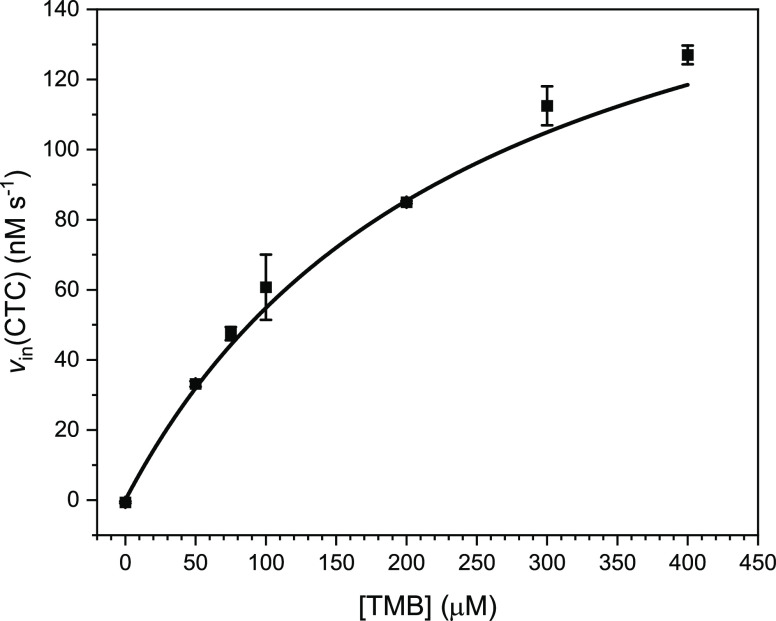
Variation of the initial rate of CTC formation from TMB on the TMB concentration at [H_2_O_2_] = 1.0 mM for the SDS/hemin/l-His system in 100 mM HEPES buffer solution (pH = 7.2) containing 5 nM hemin, 20 mM SDS and 8.0 mM l-His. The reaction was run at RT with [TMB] = 0, 50, 75, 100, 200, 300, and 400 μM; *N* = 3. The experiments were performed using the following order of addition of the corresponding stock solutions: 1. HEPES; 2. SDS; 3. Hemin; 4. l-His; 5. TMB; 6. H_2_O_2_.

For a comparison of *k*_cat,app_(H_2_O_2_) and *K*_M,app_(H_2_O_2_) of the optimal SDS/hemin/l-His system, *k*_cat_(H_2_O_2_) and *K*_M_(H_2_O_2_) were also determined for the enzyme HRPC at [HRPC] = 5 nM and [TMB] = 0.3 mM, yielding *k*_cat_(H_2_O_2_) ≈ 94.9 ± 0.4 s^–1^ and *K*_M_(H_2_O_2_) ≈ 56 ± 1 μM (see Figure S14 in the Supporting Information and Table 1). A determination of *k*_cat_(TMB) and *K*_M_(TMB) for HRPC was not possible due to the formation of the second-electron oxidation product, the TMB diamine dication (see Figure 2).

From Table 1, it is clear that HRPC is a more efficient catalyst than the optimal hemin/SDS/l-His system, having a ≈1.8-fold higher *k*_cat_(H_2_O_2_) value, a 16-fold lower *K*_M_(H_2_O_2_) value, and a *k*_cat_(H_2_O_2_)/*K*_M_(H_2_O_2_) ratio, i.e., a catalytic efficiency (specificity constant),^116,117^ which is 30 times larger than the comparative apparent value for our optimal SDS/hemin/l-His system. The difference in the catalytic efficiency between the optimal hemin system and HRPC is not unexpected. However, the obtained apparent catalytic efficiencies reported in this work are substantially higher than the previously reported efficiencies for other peroxidase-mimicking systems with hemin as a catalyst. In comparison to the values determined by Solomon et al.^99^ for a peptide-hemin system called “AHHeme,” our SDS/hemin/l-His system has an apparent catalytic efficiency (*k*_cat,app_/*K*_M,app_) which is more than 2 orders of magnitude higher (based on H_2_O_2_) and 9 orders of magnitude higher (based on TMB). A comparison to the previous work on hemin in SDS micelles by Moosavi-Movahedi et al.^55,100^ could not be made since *k*_cat,app_ and *K*_M,app_ were determined for the reducing substrate guaiacol and not for TMB or H_2_O_2_.

The observed trend of *v*_in_(CTC) vs [H_2_O_2_] in Figure 11 seems to reflect a combination of (i) initial substrate saturation behavior at low H_2_O_2_ concentrations (resembling the Michaelis–Menten kinetics of enzymes)^116,117^ and (ii) catalyst inactivation at high H_2_O_2_ concentrations. Concerning the latter point, an analysis of the CTC formation during the first 60 s for [H_2_O_2_] = 2.0–7.0 mM showed that the reaction rate started to decrease with time relatively quickly after starting the reaction, the CTC formation clearly leveling-off with time (see Figures S16 and S17A in the Supporting Information). The reason for this decrease in the CTC yield for the reactions run in excess H_2_O_2_ is unclear. There are at least two possibilities: (1) “Catalase-like activity”. If hemin follows a peroxidase-like cycle, it could be that the formed Compound I is reacting with H_2_O_2_ as a reducing substrate (and not TMB), resulting in an oxidation of H_2_O_2_ and not TMB; such a situation is known for HRP^9,120^ and (2) hemin inactivation by the formation of reactive oxygen species (ROS). In case hemin would not follow the peroxidase cycle of HRP involving a heterolytic cleavage of the O–O bond in added H_2_O_2_, but rather would catalyze a homolytic cleavage, thereby forming hydroxyl radicals (or some other types of ROS), the formed radicals could react with the labile meso-position of protoporphyrin IX, opening the ring, and releasing the iron ion, which would cause a drastic drop in catalytic activity.^121−123^ Such type of hemin degradation was discussed to occur in the case of G-quadruplex/hemin complexes.^28^ The inactivating effect of high concentrations of H_2_O_2_ on the peroxidase-like activity of the SDS/hemin/l-His system was confirmed by an additional incubation experiment, as shown in Figure S17B in the Supporting Information. Hemin (5 nM) was incubated for up to 5 min in 100 mM HEPES buffer solution (pH = 7.2) in the presence of 2.0 mM SDS, 8.0 mM l-His, and 3.0 mM H_2_O_2_. Then TMB (0.3 mM) was added, and the formation of CTC was followed for 120 s. Independent of whether the incubation of hemin was for 1, 2, 3, 4, or 5 min, *v*_in_(CTC) was only about 130 nM s^–1^ (Figure S17B in the Supporting Information) as compared to ≈250 nM s^–1^ without incubation (Figure 11). It seems that under the conditions used, about 50% of the hemin got inactivated (possibly an irreversible chemical modification of PPIX), or 2 mM of the initially present 3 mM H_2_O_2_ was consumed during the incubation time. The exact mechanism of such H_2_O_2_-induced hemin inactivation could be a part of future investigations. Obviously, the inactivation mechanism should become clearer once the catalytic steps that hemin follows during the reaction are known.

In another series of measurements, the dependence of *v*_in_(CTC) on the concentration of hemin was determined in 100 mM HEPES solution (pH = 7.2) at 2.0 mM SDS and 8.0 mM l-His with 0.3 mM TMB and 1.0 mM H_2_O_2_ (see Figure S18 in the Supporting Information). A linear dependence was found, which is typical for molecular species (i.e., enzymes) acting as catalyst in the presence of excess substrate.^116,117^

### 3.5 Use of Micelle-Forming Surfactants Different from SDS

In this part of our work, we investigated whether other micelle-forming surfactants than SDS can also have a positive effect on the peroxidase-like activity of hemin toward TMB as a reducing substrate at pH = 7.2. The surfactants used were anionic SDBS (a mixture of *para*-substituted benzenesulfonates),^124,125^ cationic CTAB, and neutral Triton X-100 (a mixture of polyethylene glycol *tert*-octylphenyl ethers) (see their chemical structures in Figure S19 in the Supporting Information). For the activity measurements, the experimental conditions were kept the same as in the case of the elaborated optimal conditions for the reaction run in the presence of SDS (without l-His): 100 mM HEPES buffer solution (pH = 7.2); [hemin] = 250 nM; [TMB] = 0.3 mM; [H_2_O_2_] = 0.3 mM, RT. For the surfactant concentrations used, we limited ourselves to 1.0, 2.0, and 4.0 mM, and *v*_in_(CTC) was determined from the change in *A*_652_ occurring during the first 60 s after the reactions were initiated by adding H_2_O_2_ (see Figures S20 and S21 in the Supporting Information). As a result, only in the case of SDBS, significant oxidation of TMB to the CTC occurred. The measured values of *v*_in_(CTC) are listed in Table 2, together with the corresponding values determined in the presence of SDS. UV–vis absorption measurements indicate that hemin in CTAB and Triton X-100 micelles are present as μ-oxo dimers.^4^ This would explain the absence of peroxidase-like activity of hemin in these two types of micelles.

**Table 2:** Comparison of the Peroxidase-like Activity of Hemin in the Presence of Different Micelle-Forming Surfactants, Measured with TMB as the Reducing Substrate at pH = 7.2

surfactant concentration (mM)	initial rate of CTC formation, *v*_in_(CTC) (nM s^–1^)^a^			
	SDS^b^	SDBS	CTAB	Triton X-100
1.0	266 ± 2	312 ± 5	0	0
2.0	308 ± 10	157 ± 13	0	0
4.0	116 ± 1	95 ± 4	0	0

a Reaction conditions: [HEPES] = 100 mM; pH = 7.2; [surfactant] = 1.0, 2.0, or 4.0 mM; [hemin] = 250 nM; [TMB] = 0.3 mM; [H_2_O_2_] = 0.3 mM; RT (see also Figure S20 in the Supporting Information).

b Data obtained from Figure 6.

### 3.6 Use of Reducing Substrates Different from TMB

Under the optimal conditions worked out for the oxidation of TMB with hemin (250 nM) as the catalyst in the presence of SDS (2.0 mM), without l-His, in 100 mM HEPES buffer solution (pH = 7.2) and H_2_O_2_ as the oxidizing substrate, we checked whether ABTS^2–^, Amplex Red, and DCFH_2_ (see Figure S22 in the Supporting Information) could also be oxidized under these conditions. The chosen substrate conditions and the determined initial rates of oxidation for these conditions are summarized in Table 3 and compared with the data for TMB. Measurements in the absence of SDS were also carried out, as well as control measurements without either hemin or H_2_O_2_ (see Figures S23–S25 in the Supporting Information).

**Table 3:** Comparison of the Peroxidase-like Activity of Hemin (250 nM) toward Different Reducing Substrates in 100 mM HEPES Buffer Solution (pH = 7.2) either in the Presence of 2.0 mM SDS or without SDS at RT

	initial rate, *v*_in_ (nM s^–1^)			
	TMB	ABTS^2–^	Amplex Red	DCFH_2_
	*v*_in_(CTC)	*v*_in_(ABTS^•–^)	*v*_in_(resorufin)	*v*_in_(DCF)
	(nM s^–1^)^a^	(nM s^–1^)^b^	(nM s^–1^)^c^	(nM s^–1^)^d^
all components	247 ± 5 (308 ± 10)^e^	149 ± 6	147 ± 4	37.5 ± 0.6
no SDS	80.3 ± 4.0 (52 ± 1)^e^	120 ± 1	139 ± 4	27.6 ± 0.3
no hemin	0	0	0	0
no H_2_O_2_	0	0	0	0

a Reaction conditions: [HEPES] = 100 mM; pH = 7.2; [SDS] = 2.0 mM; [Hemin] = 250 nM; [TMB] = 0.3 mM; [H_2_O_2_] = 0.3 mM (see Figure S9 in the Supporting Information).

b Reaction conditions: [HEPES] = 100 mM; pH = 7.2; [SDS] = 2.0 mM; [Hemin] = 250 nM; [ABTS^2–^] = 1.0 mM; [H_2_O_2_] = 0.2 mM (see Figure S23 in the Supporting Information).

c Reaction conditions: [HEPES] = 100 mM; pH = 7.2; [SDS] = 2.0 mM; [Hemin] = 250 nM; [Amplex Red] = 0.25 mM; [H_2_O_2_] = 0.25 mM (see Figure S24 in the Supporting Information).

d Reaction conditions: [HEPES] = 100 mM; pH = 7.2; [SDS] = 2.0 mM; [Hemin] = 250 nM; [DCFH_2_] = 0.05 mM; [H_2_O_2_] = 0.03 mM (see Figure S25 in the Supporting Information).

e Data from a different determination (see Figures 4, 6, and Table 2).

From the data shown in Table 3, it is clear that under the chosen conditions a peroxidase-like activity of hemin not only exists toward TMB as the reducing substrate but also toward ABTS^2–^, Amplex Red, and DCFH_2_. In all cases, the presence of 2.0 mM SDS had a positive effect on the initial rate of oxidation, although the effect in the case of ABTS^2–^, Amplex Red, and DCFH_2_ was much smaller than in the case of TMB. An interesting future study could be to investigate for each substrate the precise dependency of the initial reaction rate on the SDS concentration and to see how this dependency compares with the case of TMB, as shown in Figure 6. Is 2.0 mM SDS optimal? Is the much larger effect SDS has in the case of TMB due to more efficient binding of TMB to the micelles as compared with the other three substrates? Another question of interest is whether the observed leveling-off in the case of TMB without SDS is also observed for ABTS^2–^, Amplex Red, and DCFH_2_, and if so whether the presence of SDS eliminates this effect, as in the case of TMB. A first inspection of the time progress of the product formation shown in Figures S23A–S25A in the Supporting Information indicates that there is no (or only a weak) leveling-off in the absence or presence of SDS during the time the reaction was followed (the first 60 s) for the conditions used (different substrate concentrations in the three cases). Therefore, it seems that the interaction of the reducing substrate and/or its oxidation product with hemin causes the leveling-off in the case of TMB without SDS. This was already concluded above based on the experiments as shown in Figure S3.

For all reducing substrates used in this work at pH = 7.2, TMB, ABTS^2–^, Amplex Red, and DCFH_2_, there was no reaction without H_2_O_2_ (Table 3). This contrasts with what we observed previously in an investigation of hemin in the presence of SDBS micelles and *p*-aminodiphenylamine (PADPA) as reducing substrate, where substrate oxidation at pH = 4.3 with hemin as a catalyst also occurred without added H_2_O_2_ (in the presence of air),^46^ while no significant reaction took place at pH = 7.2 (without H_2_O_2_) (see Table 3). This indicates that the hemin-catalyzed oxidation of this type of arylamine by O_2_ present in air strongly depends on the acidity of the aqueous solution used. As mentioned in Section 1, all experiments of the present work were carried out at pH = 7.2.

### 3.7 Peroxidase-like Activity of Hemin in the Presence of SDS/Dodecanol (3:7) Vesicles

With a few preliminary experiments we wanted to find out whether hemin also shows peroxidase-like activity in the presence of SDS-based vesicles toward TMB as reducing substrate under the optimal conditions we elaborated for the presence of SDS micelles. So far, the peroxidase-like activity of hemin in vesicular systems was reported in at least two cases using either SDS and a cationic gemini surfactant,^54^ or block copolymers as vesicle-forming amphiphiles.^126^ The use of vesicles instead of micelles is of interest for further developing scenarios about the possible role of hemin or other metalloporphyrins as simple catalysts in prebiological cell-like molecular assemblies. Vesicles with their trapped aqueous volume and a boundary of amphiphilic molecules currently are considered as reasonable models of prebiological compartment systems,^127−136^ although nobody knows how the first cells emerged from nonliving chemical systems at the origin of life.^137^ Micelles^138^ or coacervates^135,139^ are another type of potentially prebiotic aggregates that may have promoted the progression of reactions in a hypothetical mixture of prebiotic compounds undergoing chemical transformations in a network of protometabolic reactions, possibly involving molecular self-replication and compartment reproduction.^136,140^

**Figure 13:**
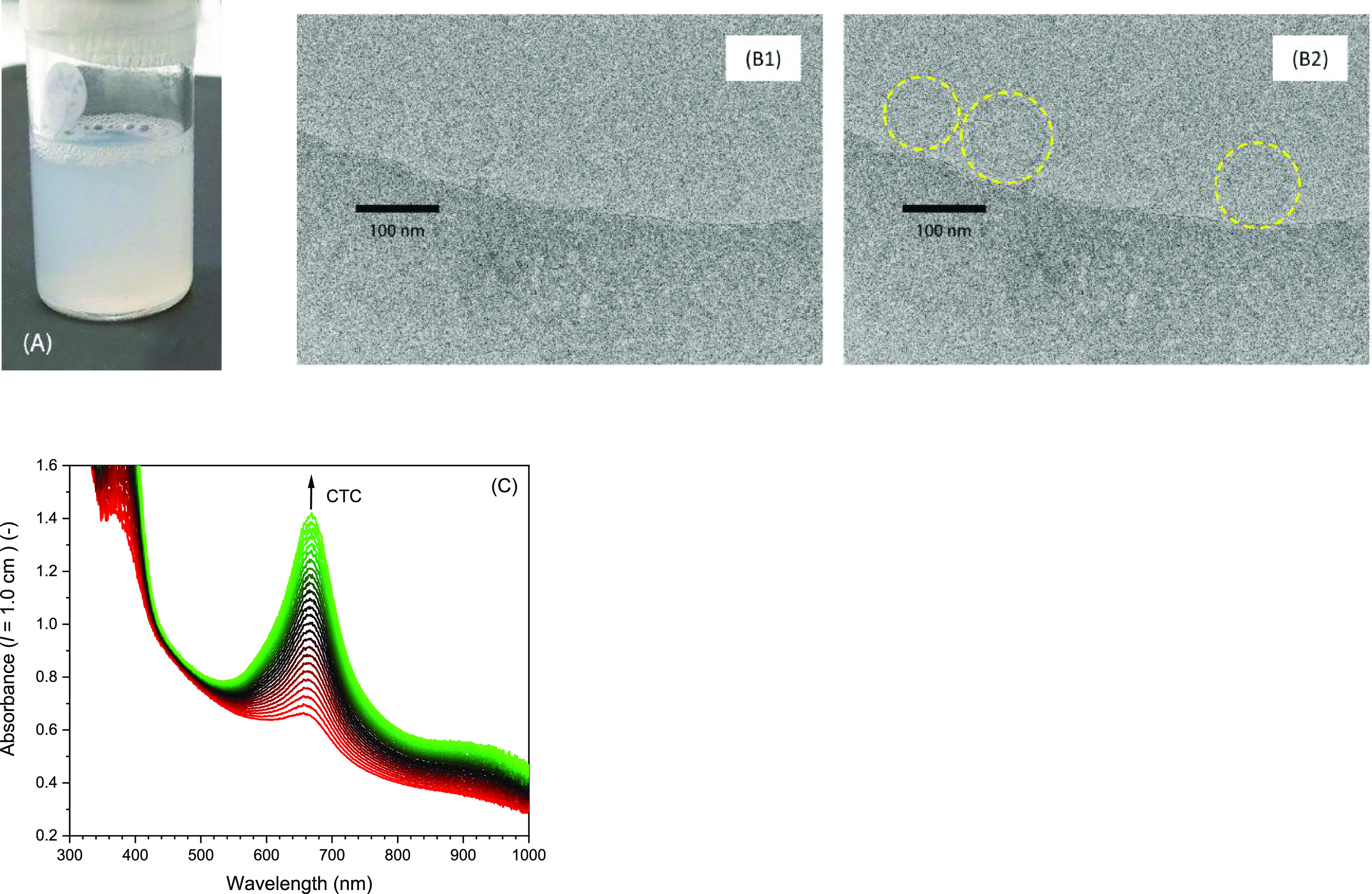
(A) Photograph of a dispersion of SDS/dodecanol (3:7, mol ratio) vesicles, prepared in 100 mM HEPES buffer solution (pH = 7.2) at [SDS] + [dodecanol] = 50 mM using the polycarbonate membrane extrusion method, see Supporting Information. (B) Cryo-TEM image indicates the presence of large unilamellar SDS:dodecanol (3:7) vesicles. (B1) Contrast of the image was enhanced in an attempt for better recognizing the two vesicles localized above the scale bar. The darker lower part of the image is the holey carbon film. (B2) Since a better contrast could not be achieved, the presence of the vesicles is indicated with dashed circles. (C) Peroxidase-like activity of hemin in a SDS:dodecanol (3:7) vesicle dispersion toward TMB as reducing substrate. Reaction conditions: [HEPES] = 100 mM; pH = 7.2; [SDS] + [dodecanol] = 6.25 mM ([SDS]/[dodecanol] = 3:7); [hemin] = 250 nM; [TMB] = 0.3 mM; [H_2_O_2_] = 0.3 mM; RT. The change of the UV–vis absorption spectrum of the reaction mixture with time is shown, as measured every 2 s for a total of 50 s.

The vesicles we used were prepared by polycarbonate membrane extrusion from SDS and dodecanol at a molar ratio of 3:7 in 100 mM HEPES buffer solution (pH = 7.2) and a total concentration of SDS + dodecanol of 50 mM; see Supporting Information. The dispersion obtained was translucent and colloidally stable for at least 16 h at RT (see Figure 13A). The existence of large unilamellar vesicles of about 100 nm was confirmed by cryogenic transmission electron microscopy (cryo-TEM) (see Figure 13B). In Figure 13C, the peroxidase-like activity of hemin (250 nM) in a vesicle dispersion of [SDS] + [dodecanol] = 6.25 mM ([SDS] = 1.88 mM, [dodecanol] = 4.37 mM) toward TMB (0.3 mM) as a reducing substrate with 0.3 mM H_2_O_2_ as oxidizing substrate at RT is shown, as experimentally determined by monitoring the time-dependent change in the UV–vis absorption spectrum of the reaction mixture. For these conditions, *v*_in_(CTC) was determined to ≈365 nM s^–1^ (see Figure S26B in the Supporting Information). The batch-to-batch variation of *v*_in_(CTC) was between 293 ± 7 nM s^–1^ and 490 ± 17 nM s^–1^ (see Figure S27 in the Supporting Information). Although the turbidity of the vesicle dispersion caused light scattering, the formation of the CTC as the reaction product is very clear. For the reference measurements without vesicles (*v*_in_(CTC) ≈95 nM s^–1^), similar to the value shown in Figure 6), and for two control measurements without hemin (*v*_in_(CTC) ≈5 nM s^–1^) or without H_2_O_2_ (*v*_in_(CTC) ≈15 nM s^–1^) (see Figure S26 in the Supporting Information).

The determined rate of CTC formation for hemin in 100 mM HEPES buffer solution (pH = 7.2) in the presence of SDS/dodecanol vesicles of *v*_in_(CTC) was ≈365 nM s^–1^ (Figure S26B in the Supporting Information), slightly higher than the value determined for the optimal SDS concentration in the micellar system in the same buffer solution (≈308 nM s^–1^, Figure 6). Efforts to further increase *v*_in_(CTC) in the case of the vesicle system by adding l-His yielded *v*_in_(CTC) ≈ 850 nM s^–1^ for [l-His] = 20 mM (see Figure S28 in the Supporting Information). This value is very similar to the one determined for the SDS micellar system and [l-His] = 8.0 mM (see Figure 9A). The micellar and vesicular systems differ in their molecular composition and the aggregation state (2.0 mM SDS in the case of the micellar solution, and 1.88 mM SDS in the case of the vesicular dispersion containing dodecanol as additional bilayer-forming compound). The proposed binding of HEPES and SDS to hemin as well as the coordination of l-His to hemin in these two systems certainly differ in their association and complex formation constants. Nevertheless, the peroxidase-like activity of hemin in the two systems was very similar.

## 4 Prebiological Considerations

The catalytic activity of apoprotein-free hemin—or other types of iron porphyrins—in an aqueous medium is of interest not only for accelerating certain biomimetic redox reactions under environmentally friendly conditions^56^ but also for a scenario about the possible prebiological presence of iron porphyrins and their possible role as catalysts on the early Earth, before the first living cells emerged. The following are some considerations concerning such a scenario and the relevance of the experimental results we obtained for these scenarios.

Currently, there is no general agreement about whether porphyrins and iron porphyrins—or other metal porphyrins—were already present on the early Earth, before life originated,^141^ or even whether the last universal common ancestor (LUCA) contained iron porphyrin-based enzymes.^141^ There are contradicting conclusions drawn from the elaborated heme biosynthesis pathways and phylogenetic analyses.^142^ On the one hand, Lane and Martin^143^ suggest that the LUCA most likely did not contain proteins consisting of iron porphyrins because it is assumed that there was no need for (heme-containing) catalysts accelerating the removal of ROS since they were considered absent on the early Earth with a reducing ancestral atmosphere (dominated by CO_2_, H_2_O vapor, N_2_, and traces of CH_4_).^144^ On the other hand, Neubeck and Freund^145^ argue on the basis of a phylogenetic analysis^146^ that the LUCA must have contained a catalase (an enzyme, which—at least today—consists of ferric heme *b* (= hemin) as a prosthetic group) and other ROS-degrading enzymes (possibly a peroxidase).^144,145,147^ Interestingly, a contemporary catalase of the fungus *Aspergillus niger* was also found to catalyze the decomposition of reactive sulfur species (RSS), such as H_2_S_2_ (which may have formed in prebiotic times from H_2_S),^148^ supporting the suggestion of an early importance of enzymes with catalase activities. Although probably not abundant in the early atmosphere, H_2_O_2_ and O_2_ might have been formed on the surface of the early Earth,^149^ arguing that not only the chemical composition of the atmosphere should be considered but also the likely heterogeneous distribution of chemical compounds on the Earth’s surface.^144^ Various studies have shown that H_2_O_2_ easily forms from H_2_O on the surface of minerals, for example, pyrite (FeS_2_)^150,151^ or silicates (SiO_2_),^152^ or on the surface of micrometer-sized water droplets,^153^ through the intermediate formation of hydroxyl radials (HO^•^).

Although there are several reports on the potentially prebiotic synthesis of porphyrins,^154−156^ porphyrins are considered as biomarkers (“biosignatures”),^157,158^ i.e., if detected on other planets than the Earth in the solar system or on extrasolar planets would be taken as sign of extraterrestrial life forms. If porphyrins formed on the early Earth, before life originated, metalation of the porphyrins would probably have happened relatively easily,^159,160^ i.e., iron porphyrins similar to heme *b* would have formed due to the large abundance of iron ions on the prebiotic Earth. Ferrous iron, Fe(II), could have been oxidized photochemically to ferric iron, Fe(III).^161^

Among the prebiotically plausible syntheses of porphyrins,^154−156^ the work of Alexy et al.^156^ is worth mentioning. The authors showed with laboratory experiments that 2,3,7,8,12,13,17,18-octaethylporphyrin forms in high yield (≈30%) within a few hours from 3,4-diethylpyrrole and formaldehyde at 50 °C in the presence of anionic SDS micelles. Reaction products were also obtained in the presence of cationic micelles (formed from hexadecyltrimethylammonium chloride; ≈20% yield at 50 °C).^156^ Since no porphyrin reaction products were obtained in the absence of surfactants, it is likely that the reaction took place in the region of the micelles, although, details of the reaction mechanism were not yet studied.^156^ The use of micelles (or vesicles) for promoting chemical reactions is known from a lot of previous investigations.^109,110,162^ Although it is generally accepted that organic molecules with amphiphilic properties and capabilities of self-assembling into aggregates (micelles or bilayered vesicles) were present on the early Earth (most likely short- and medium-chain fatty acids),^163^ molecules like SDS generally are not considered prebiotic compounds. However, a recent analysis of the soluble part of the organic matter of the Murchison meteorite seems to demonstrate a rich abundance of alkylsulfonates (including dodecylsulfonate, C_12_H_25_SO_3_^–^) and alkylbenzenesulfonates in such carbonaceous meteorites.^164^ A previous analysis of the *insoluble organic matter* of this type of meteorite^165^ is in qualitative support of the mentioned recent findings. Therefore, it is possible that aggregate-forming alkylsulfonates and sulfates were delivered to the early Earth during the second wave of meteorite bombardment,^166^ before any life forms existed. Although SDS was used in our experiments on the catalytic activity of hemin and in the experiments carried out by Alexy et al. on the synthesis of 2,3,7,8,12,13,17,18-octaethylporphyrin,^156^ it is likely that a micelle-forming alkylsulfonate would also support the reactions in a similar way as SDS. In the case of the catalytic activity of hemin, SDBS was already successfully used for promoting and guiding the oxidation of PADPA.^56^

As a final point, l-His was used in our work as an activity enhancer of hemin. According to Vázquez-Salazar et al.,^167^l-His is not a prebiotically plausible compound. Neither its synthesis under prebiotically plausible conditions has been demonstrated^167^ nor it was found in extracts of a carbonaceous meteorite.^168^ Other prebiotically plausible electron pair-donating small molecules, e.g., adenine, may, however, have the same effect on hemin as l-His had in our work.

In conclusion, it is possible that iron porphyrins already played a role in prebiotic times on the early Earth, possibly as ROS (and RSS)-degrading enzyme-like systems formed with amphiphile aggregates, well before the suggested formation of the more complex hemin-G-quadruplex RNA structures.^26b,169^ Obviously, micelle-forming amphiphiles are chemically much simpler than oligonucleotides with a specific sequence.

## 5 Summary and Outlook

**Figure 14:**
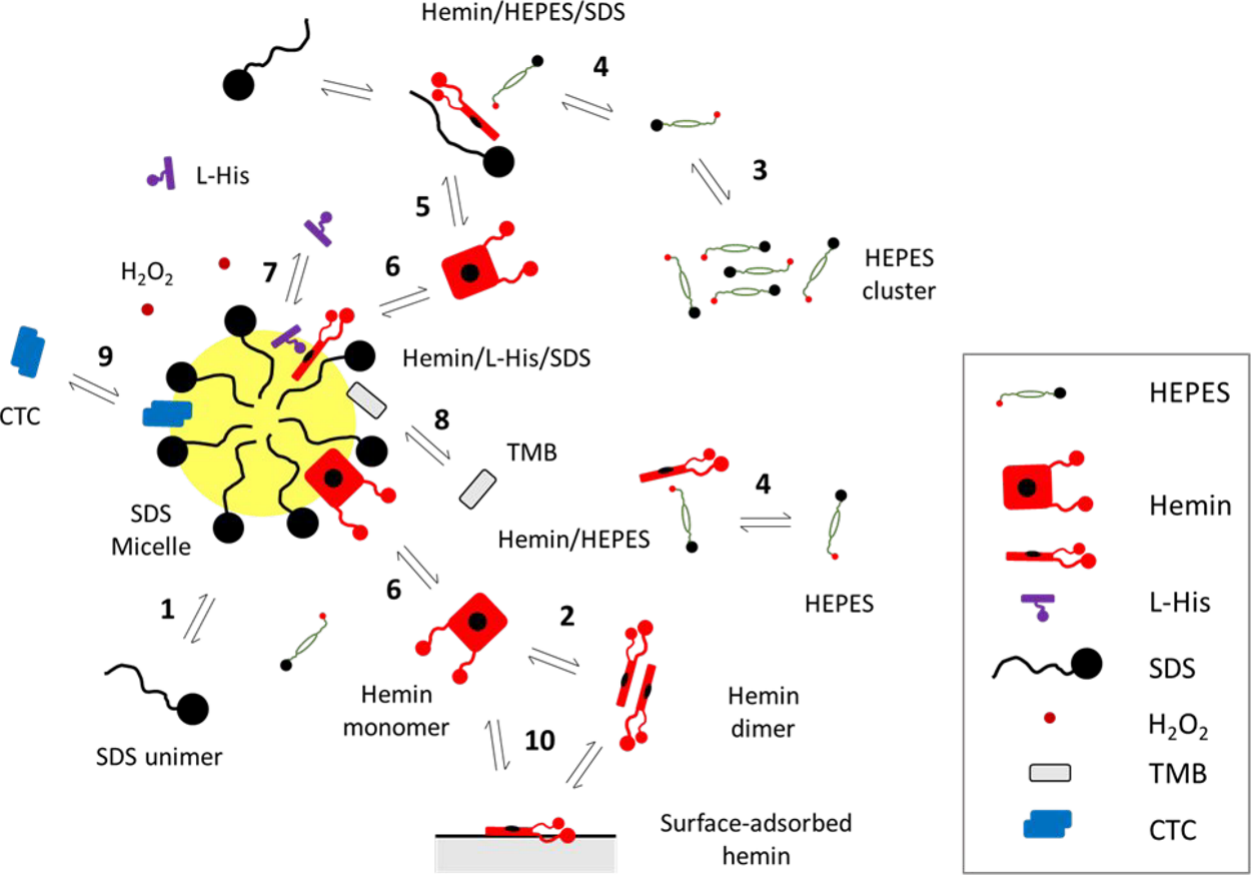
Schematic representation of possible interactions and molecular states in an aqueous mixture of HEPES, SDS (at a concentration around the cmc), hemin, l-His, TMB, and the CTC product formed from TMB upon addition of H_2_O_2_ with hemin as the catalyst (peroxidase-like activity). The key features considered are (1) existence of SDS micelles and SDS unimers (or small SDS aggregates); (2) existence of hemin monomers and dimers (and possibly higher aggregates); (3) formation of “HEPES clusters”; (4) interaction of HEPES with hemin; (5,6) interaction of SDS with hemin; (7) interaction of l-His with hemin; and (8,9) interactions of TMB and the CTC with SDS micelles (and possibly unimers). (10) Hemin can also be adsorbed on the wall of the vessel, in which the reaction mixtures are investigated.

Despite numerous previous studies on the effect of micelles on the aggregation state of hemin in an aqueous solution,^4,25,26a,43−46,48,49^ there is still only a limited number of studies addressing the catalytic activity of hemin in the presence of micelle-forming surfactants.^50,53−57^ Our work is a contribution to a better understanding of the influence of HEPES, the micelle-forming surfactant SDS, and l-His on the peroxidase-like activity of hemin in an aqueous solution near neutral pH (pH = 7.2). One reason for the lack of literature data can be seen in the complexity of the reaction mixtures of interest, where, for example, a variation of the SDS content leads to drastic changes in the physicochemical properties of the system (formation of micellar aggregates above the cmc). There are several intermolecular interactions that should be considered, as they can significantly affect the catalytic performance of hemin in this type of system (see Figure 14).

Although it is not straightforward to determine the individual contributions of the various interactions to the measured catalytic activity of hemin, the following conclusions can be drawn from our work using mainly TMB as the reducing substrate at [hemin] = 250 nM and [TMB] = [H_2_O_2_] = 0.3 mM (pH = 7.2).

1. The presence of HEPES has a positive effect on the initial rate of CTC formation, *v*_in_(CTC), as well as on the reaction yield but without achieving high yields due to reversible inactivation (possibly by product inhibition) (Figures 4 and S3 in the Supporting Information). We have no evidence that this effect is somehow related to the propensity of HEPES to form radicals in the presence of H_2_O_2_.^170^ The positive effect of HEPES seems to be due to binding of HEPES to Fe(III), as indicated by MD simulations (Figure 5B). Such binding may increase the electron density at Fe(III), thereby accelerating the release of an electron from Fe(III) during the reaction with H_2_O_2_ (Fe(III) oxidation), without significantly altering the aggregation state of hemin (Figure 5A).
2. SDS has a positive effect on *v*_in_(CTC) and the reaction yield already at SDS concentrations below the cmc, with the highest activity at [SDS] ≈ cmc (≈6-fold increase in *v*_in_(CTC) at 100 mM HEPES) (Figure 6). UV–vis absorption measurements indicate that the addition of SDS to hemin in aqueous HEPES solution increases the relative amount of nonaggregated hemin. Interactions between the dodecyl chain of SDS and the hydrophobic part of PPIX were demonstrated by MD simulations, see Figure 8 as well as Movies S1 and S2 in the Supporting Information. In the presence of SDS, hemin inactivation did not occur. Inactivated hemin in the absence of SDS was reactivated after the addition of micellar SDS (Figure S3 in the Supporting Information).
3. The activity of hemin at [SDS] ≈ cmc is increased in the presence of l-His up to ≈2.5-fold at 8.0 mM l-His (for 2.0 mM SDS at 100 mM HEPES) (Figure 9). UV–vis absorption measurements (Figure 10) indicate that l-His coordinates to Fe(III) in a l-His concentration-dependent manner, with the formation of more and more bis-l-His-ligated hemin above 8.0 mM l-His, which would explain the observed decrease in *v*_in_(CTC) above this optimal l-His concentration.
4. With TMB as a reducing substrate, the presence of SDBS instead of SDS (both with an anionic headgroup) also leads to an increase in *v*_in_(CTC), while no hemin activity could be measured in the presence of CTAB micelles (cationic) or triton X-100 micelles (nonionic) (Table 2), indicating the importance of the chemical structure of the surfactant, at least for the type of reducing substrate and the low amount of hemin (250 nM) used.
5. For reactions investigated in the presence of SDS (2.0 mM), a peroxidase-like activity could also be detected with ABTS^2–^, Amplex Red, or DCFH_2_ as reducing substrates instead of TMB. However, in the three cases, there was no big difference between the activity in the presence of SDS and without SDS (Table 3), possibly reflecting differences in reducing substrate type–micelle interactions (stronger in the case of TMB as compared to the other three substrates).
6. Although a fair comparison with related synthetic peroxidase-mimicking systems is challenging, the SDS/hemin/l-His mixture we have investigated in HEPES buffer solution showed a remarkably high TMB substrate turnover (*k*_cat,app_ (TMB) = 39 ± 3 s^–1^) if used at the optimal conditions: 100 mM HEPES; pH = 7.2; 2.0 mM SDS; 5.0 nM hemin; 8.0 mM l-His, measured with 0.3 mM TMB and 1.0 mM H_2_O_2_ at RT (see Section 3.4, Table 1).

Despite the different results obtained in our fundamental investigation, there are at least two questions that would be worth addressing in future studies:

1. Which are the catalytic steps of hemin in this non-natural system (i.e., which is the reaction mechanism)? Are Compound I and Compound II formed like in the case of a heme peroxidase (see Section 1)?
2. What is a good model to visualize the average binding of hemin to an SDS micelle under the conditions yielding the highest peroxidase-like activity?

**Figure 15:**
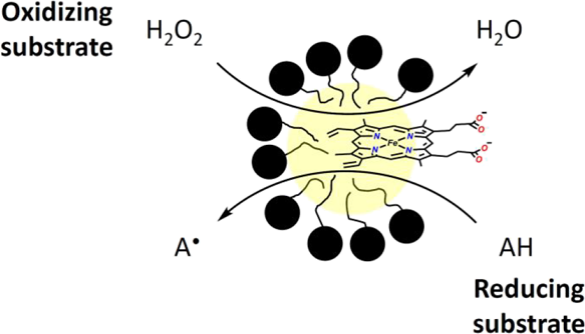
Oversimplified illustration of the binding of hemin to an SDS micelle considering the amphiphilic nature of hemin, but *without* taking into account specific interactions that seem to exist between HEPES and hemin, SDS and hemin as well as between added l-His and hemin. If a hemin-catalyzed reaction occurs according to the peroxidase cycle of heme peroxidases, hemin is first oxidized in a two-electron oxidation reaction by H_2_O_2_ to Compound I (por^+•^)Fe^IV^(O), which then leads to two consecutive one-electron oxidations of two molecules of a reducing substrate (AH) to yield two molecules of radical A^•^, which may further react uncatalyzed (see Section 1). Modified from Cvjetan, N.; Walde, P. Ferric heme *b* in aqueous micellar and vesicular systems: state-of-the-art and challenges. *Q. Rev. Biophys.***2023**, 56, e1, 1–43. Copyright 2023 of the authors. Published by Cambridge University Press.^4^

As for the second question, it is likely that the iron ion of hemin and ligated l-His prevent deep embedding of hemin in the hydrophobic core of the micelle. The drawing in Figure 15 is certainly an overly simple illustration of the real situation. Hemin is more likely to be localized to the surface of the micelle. If this were the case, how would the situation differ in the case of micelles from SDBS?

The fact that hemin shows peroxidase-like activity also in dispersions of SDS/dodecanol (3:7, mole ratio) vesicles is of interest with respect to scenarios about the possible emergence of life from prebiological cell-like compartment systems at the origin of life. Vesicles of chemically simple amphiphiles are one type of such compartment systems,^128−136,140^ although it is not completely clear whether the SDS used in our work can be considered a prebiotic compound (see Section 4). Furthermore, PPIX—with two propionic acids, two vinyl groups, and four methyl groups at defined positions on the porphyrin ring (Figure 1A)—was likely absent in prebiotic times; however, it has been shown that chemically simpler porphyrins can be synthesized in the laboratory under potentially prebiotic conditions and that their yield was significantly increased when micelles or vesicles were added.^156^ Such porphyrins, free or metallized, may have been associated with membranous structures (such as vesicles) and acted as primitive catalysts to promote and control prebiotic chemical transformations, contributing to protometabolic reaction networks that may have existed on early Earth before the first living cells emerged.

In addition to these pure speculations, stable and catalytically active surfactant/hemin-based assemblies (vesicles or micelles) may find applications as cheap peroxidase-mimicking systems for catalyzing chemical transformations for analytical or synthetic applications.^56,57^

The developed hemin-SDS-l-His system with TMB as a reducing substrate could be utilized directly or with some modifications if required for various applications. One example is the detection of hemin or hydrogen peroxide as an alternative method to the ones reported in the literature.^171−174^ Our system appears to be advantageous in terms of simplicity of the preparation procedure and cost-efficiency. As reported in this work, hemin concentrations as low as 2 nM can be detected, although we did not investigate the limit of detection. With some modifications, the system described could probably also be applied for the detection and determination of glucose.^175,176^ Finally, synthetic applications of the hemin-SDS-l-His system could also be possible for all those cases where hemin was shown previously to act as a catalyst, for example, for the transformation of nitrosamines^177^ (water pollutants), the oxidative cyanation of secondary amines,^178^ and sulfonium ylide formation.^179^ Interestingly, hemin-catalyzed reactions can also occur under aerobic conditions in the absence of a peroxide.^180−182^ It remains to be investigated whether the system reported in this work could be applied to those reactions or similar reactions.

## Supporting Information Available

Data on the peroxidase-like activity of hemin in different HEPES buffer solutions, measured with TMB and H_2_O_2_ as substrates; on the cmc determination of SDS; on the effect of SDS and l-His on the peroxidase-like activity of hemin using TMB as a reducing substrate; on the effect of SDBS, CTAB or Triton X-100 on the peroxidase-like activity of hemin using TMB as a reducing substrate; on the use of ABTS^2–^, Amplex Red, and DCFH_2_ as reducing substrates; on the preparation of SDS/dodecanol (3:7) vesicles and their effect on the peroxidase-like activity of hemin with TMB as a reducing substrate; and on MD simulations (PDF)
MD simulations (150 ns) indicating persistence of the interactions (MP4)
MD simulation of a box (3,000 nm^3^) containing 180 HEPES molecules (MP4)

## Abbreviations

ABTS^2–^: 2,2′-azino-bis(3-ethylbenzothiazoline-6-sulfonate)
cmc: critical micellization concentration
CTAB: cetyltrimethylammonium bromide
CTC: charge transfer complex
DCF: 2,7-dichlorofluorescein
DCFH_2_-DA: 2,7-dichlorodihydrofluorescein diacetate
DCFH_2_: 2,7-dichlorodihydrofluorescein
DMSO: dimethyl sulfoxide
HEPES: 4-(2-hydroxyethyl)-1-piperazineethanesulfonic acid
HRP: horseradish peroxidase
HRPC: horseradish peroxidase isoenzyme C
MD: molecular dynamics
PAPDA: *p*-aminodiphenylamine
PPIX: protoporphyrin IX
RT: room temperature
SDS: sodiumdodecyl sulfate
SDBS: sodium dodecylbenzenesulfonate
TMB: 3,3′,5,5′-tetramethylbenzidine
