# Supplementary material for: Optimization and Enhancement of the Peroxidase-like Activity of Hemin in Aqueous Solutions of Sodium Dodecylsulfate
Source: ACS Omega. 2023 Nov 3;8(45):42878–99. doi: 10.1021/acsomega.3c05915 (PMC10652838; doi:10.1021/acsomega.3c05915)
Supplement: Supplementary file 1 — ao3c05915_si_001.pdf [file ao3c05915_si_001.pdf]

Supporting Information

Optimization and Enhancement of the Peroxidase-like Activity of Hemin in Aqueous Solutions of Sodium Dodecylsulfate (SDS)

Nemanja Cvjetan,[†] Lukas D. Schuler,[‡] Takashi Ishikawa,[±] and Peter Walde*[,†]

[†]*Department of Materials, ETH-Zürich, Leopold-Ruzicka-Weg 4, 8093 Zürich, Switzerland*

[‡] *xirrus GmbH, Buchzelgstrasse 36, 8053 Zürich, Switzerland*

[±] *Department of Biology and Chemistry, Paul Scherrer Institute and Department of Biology, ETH-Zürich, Forschungsstrasse 111, 5232 Villigen PSI, Switzerland*

*Corresponding author

Content

1. Reaction of Hemin in HEPES Buffer Solutions Using TMB as Reducing Substrate	**Figure S1**	p. S3
2. MD Simulations of HEPES Molecules in Water at pH = 7.2	**Figure S2**	p. S4
3. Reactivation of Hemin in HEPES Buffer Solution by Adding SDS	**Figure S3**	p. S5
4. Determination of the cmc of SDS With Pinacyanol Chloride	**Figure S4**<br>**Figure S5**<br>**Figure S6**<br>**Figure S7**	p. S6
5. Effect of SDS on the Peroxidase-like Activity of Hemin Using TMB as Reducing Substrate	**Figure S8**<br>**Figure S9**	p. S9
6. Effect of SDS on the Stability of Aqueous Hemin Solutions Kept Inside Polystyrene Cuvettes	**Figure S10**	p. S11
7. Effect of L-His on the Peroxidase-like Activity of Hemin in the Presence and Absence of SDS Using TMB as Reducing Substrate	**Figure S11**<br>**Figure S12**<br>**Figure S13**<br>**Figure S14**	p. S12
8. Determination of $K_M(H_2O_2)$ and $k_{cat}(H_2O_2)$ of HRP Measured with TMB as Reducing Substrate	**Figure S15**	p. S15
9. Effect of H_2O_2 on the Peroxidase-like Activity of Hemin/SDS/L-His Using TMB as Reducing Substrate	**Figure S16**<br>**Figure S17**	p. S16
10. Effect of the Hemin Concentration on the Peroxidase-like Activity of Hemin/SDS/L-His Using TMB as Reducing Substrate	**Figure S18**	p. S18
11. Effect of the Presence of SDBS, CTAB, or Triton X-100 on the Peroxidase-like Activity of Hemin in HEPES Buffer Solution at pH = 7.2, Measured with TMB as Reducing Substrate	**Figure S19**<br>**Figure S20**<br>**Figure S21**	p. S19
12. Peroxidase-like Activity of Hemin in HEPES Buffer Solution (pH = 7.2) in the Presence of SDS Against $ABTS^{2-}$, Amplex Red, or $DCFH_2$ as Reducing Substrates	**Figure S22**<br>**Figure S23**<br>**Figure S24**<br>**Figure S25**	p. S21
13. Experiments with Vesicle Dispersions Prepared from SDS and Dodecanol in HEPES Buffer Solution (pH = 7.2)	**Figure S26**<br>**Figure S27**<br>**Figure S28**	p. S24
14. References		p. S28
15. Contents of the Supporting Movies	**Movie S1**<br>**Movie S2**	p. S30

1. Reaction of Hemin in HEPES Buffer Solutions Using TMB as Reducing Substrate

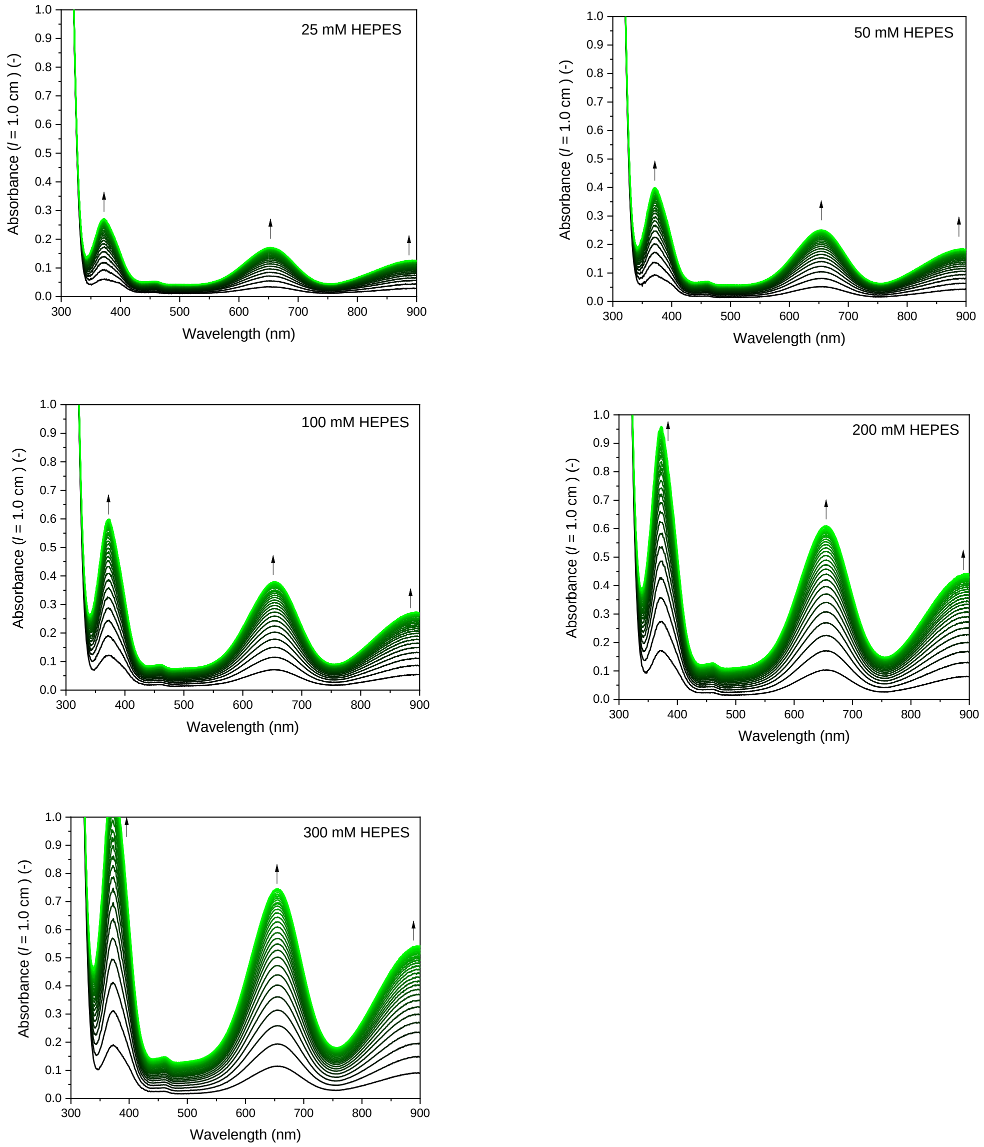

Figure S1. Analysis of the hemin-catalyzed oxidation of TMB in different HEPES buffer solutions. Development of the UV−vis absorption spectra of the reaction mixtures over time for different concentrations of HEPES. *Reaction conditions:* [HEPES] = 25, 50, 100, 200, or 300 mM; pH = 7.2; [hemin] = 250 nM; [TMB] = 0.3 mM; [H_2O_2] = 0.3 mM; RT. The spectra were recorded every 10 s for a duration of 300 s.

2. MD Simulations of HEPES Molecules in Water at pH = 7.2

(A) (B)

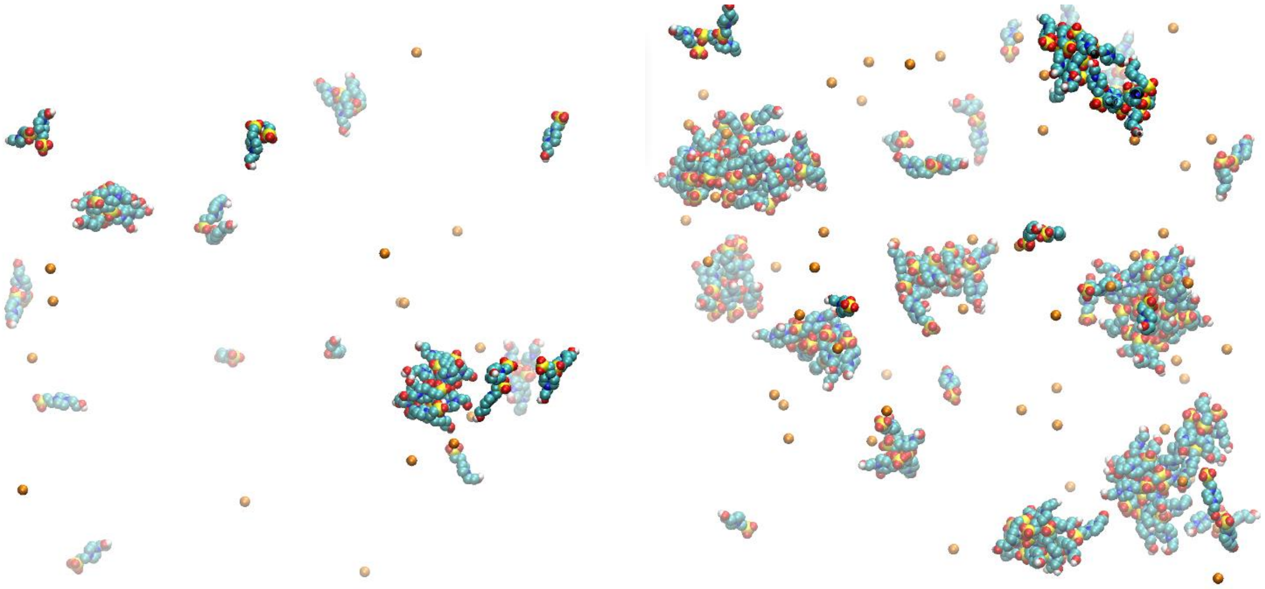

Figure S2. Snapshot of the MD simulation of 25 M (A) and 100 M (B) HEPES at pH = 7.2 after 50 ns; the water molecules are not shown. The simulations started with a random placement of the HEPES molecules. Color code: white, H-atom; cyan, C-atom; red, O-atom; yellow, S-atom; blue, N-atom; orange, Na^+ ion. The simulation box (3,000 nm^3) contained either 45 HEPES molecules (25 M) or 180 HEPES molecules (100 M). Note that simulations of 25 or 100 mM HEPES were not possible with the method and volume of the simulation box used, as there would be less than one molecule per box.

3. Reactivation of Hemin in HEPES Buffer Solution by Adding SDS

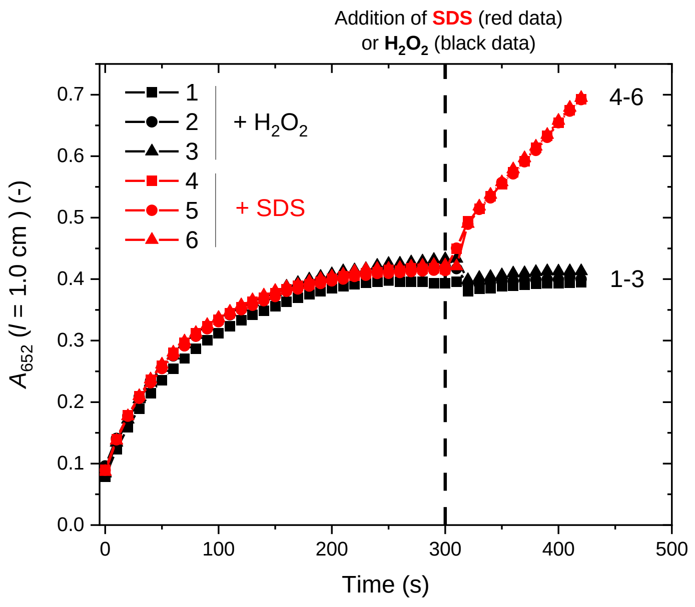

Figure S3. Reactivation of hemin in a reaction mixture consisting of hemin, TMB, H_2O_2, and HEPES after the reaction leveled-off after 300 s. *Reaction conditions:* [HEPES] = 100 mM; pH = 7.2; [hemin] = 250 nM; [TMB] = 0.3 mM; [H_2O_2] = 0.3 mM; RT. After 5 min of reaction, either a new portion of H_2O_2 (300 µM, black data points, 1-3) or a new portion of SDS (2.0 mM, red data points, 4-6) was added. The absorption spectrum of the reaction mixture was recorded every 10 s for a total of 7 min (number of measurements, N = 3). The formation of the CTC is shown by plotting A_{652} *vs.* time. *Result:* Only after addition of SDS the reaction proceeded beyond 5 min.

4. Determination of the cmc of SDS With Pinacyanol Chloride

The cmc of SDS was determined following published protocols.[S1-S4] In short, UV−vis spectral changes of pinacyanol chloride were followed with variation of the concentration of SDS. The cmc value was determined in HEPES buffer solution (25 mM, 50 mM, or 100 mM), pH = 7.2, either (i) in the absence or (ii) in the presence of hemin (250 nM) and TMB (300 µM). This comparison was made to find out whether the presence of hemin and TMB affect the cmc value of SDS. The total concentration of pinacyanol chloride was always 5.0 µM, and the total volume 1.0 mL. All spectra were recorded at T = 25 °C with a JASCO V-670 UV−vis−NIR spectrophotometer (using a quartz cuvette of pathlength 1.0 cm). The cmc value was taken as the SDS concentration at which with increasing SDS concentration A_{606} started to increase (indicating initiation of pinacyanol chloride binding to the first micelles formed).

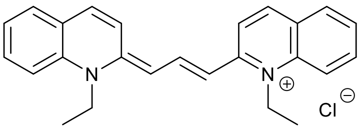

Pinacyanol chloride

As shown in **Figures S4 – S6**, the cmc of SDS showed dependence on the HEPES concentration and the presence of hemin and TMB resulted in a lowering of the cmc values. For a comparison, the cmc was also determined for a 100 mM sodium phosphate buffer solution, see **Figure S7A**. The peroxidase-like activity of hemin against TMB in the presence of the phosphate buffer solution was found to be much lower than in the presence of the HEPES buffer solution of the same pH = 7.2, see **Figure S7B**.

In 100 mM HEPES buffer solution, pH = 7.2

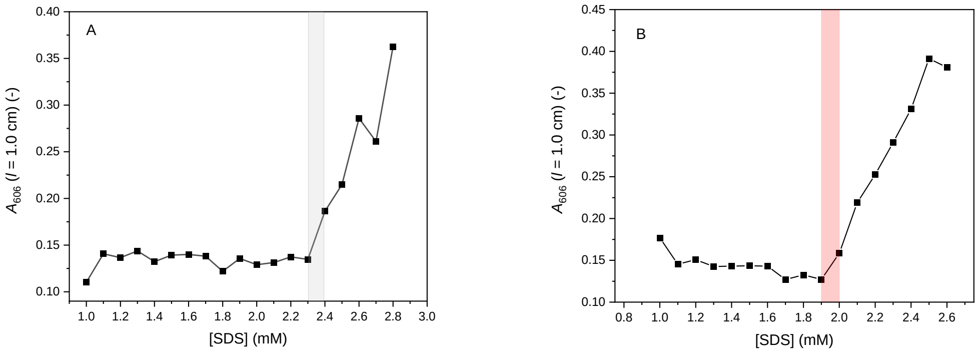

Figure S4. Determination of the cmc of SDS in *100 mM HEPES* buffer solution at T = 25 °C. **(A)** Conditions: pH = 7.2; [SDS] = 1.0 – 2.6 mM (in 0.1 mM steps); [pinacyanol chloride] = 5 µM; *no* hemin; *no* TMB. *Result:* cmc = 2.3 – 2.4 mM. **(B)** Determination of the cmc of SDS in *100 mM HEPES* buffer solution. Conditions: pH = 7.2; [SDS] = 1.0 – 2.6 mM (in 0.1 mM steps); [pinacyanol chloride] = 5 µM; [hemin] = 250 nM; [TMB] = 0.3 mM; 0.85 vol% DMSO. *Result:* **cmc = 1.9 – 2.0 mM**.

In 50 mM HEPES buffer solution, pH = 7.2

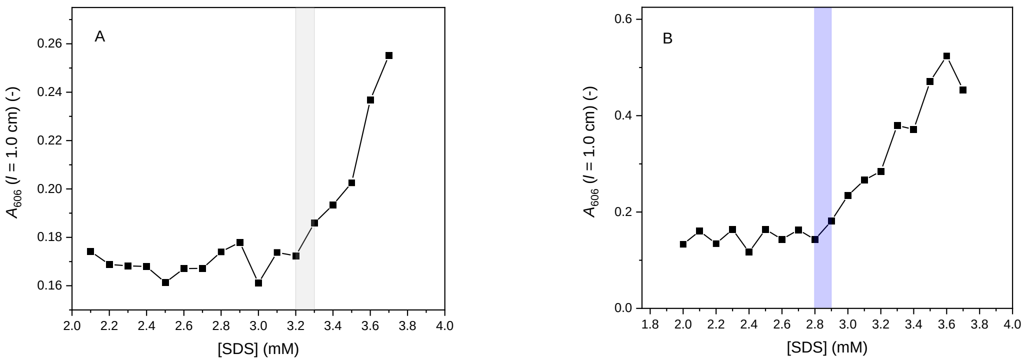

Figure S5. Determination of the cmc of SDS in *50 mM HEPES* buffer solution at T = 25 °C. **(A)** Conditions: pH = 7.2; [SDS] = 2.1 – 3.7 mM (in 0.1 mM steps); [pinacyanol chloride] = 5 µM; *no* hemin; *no* TMB. *Result:* cmc = 3.2 – 3.3 mM. **(B)** Conditions: pH = 7.2; [SDS] = 2.0 – 3.7 mM (in 0.1 mM steps); [pinacyanol chloride] = 5 µM; [hemin] = 250 nM; [TMB] = 0.3 mM; 0.85 vol% DMSO. *Result:* **cmc = 2.8 – 2.9 mM**.

In 25 mM HEPES buffer solution, pH = 7.2

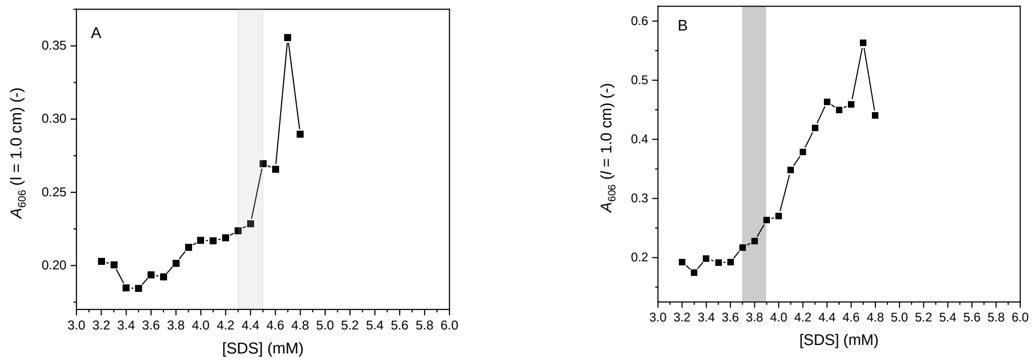

Figure S6. Determination of the cmc of SDS in *25 mM HEPES* buffer solution at T = 25 °C. **(A)** Conditions: pH = 7.2; [SDS] = 3.2 – 4.8 mM (in 0.1 mM steps); [pinacyanol chloride] = 5 µM; *no* hemin; *no* TMB. *Result:* cmc = 4.3 – 4.5 mM. **(B)** Conditions: pH = 7.2; [SDS] = 3.2 – 4.8 mM (in 0.1 mM steps); [pinacyanol chloride] = 5 µM; [hemin] = 250 nM; [TMB] = 0.3 mM; 0.85 vol% DMSO. *Result:* **cmc = 3.7 – 3.9 mM**.

In 100 mM sodium phosphate buffer solution, pH = 7.2 (cmc and activity determinations)

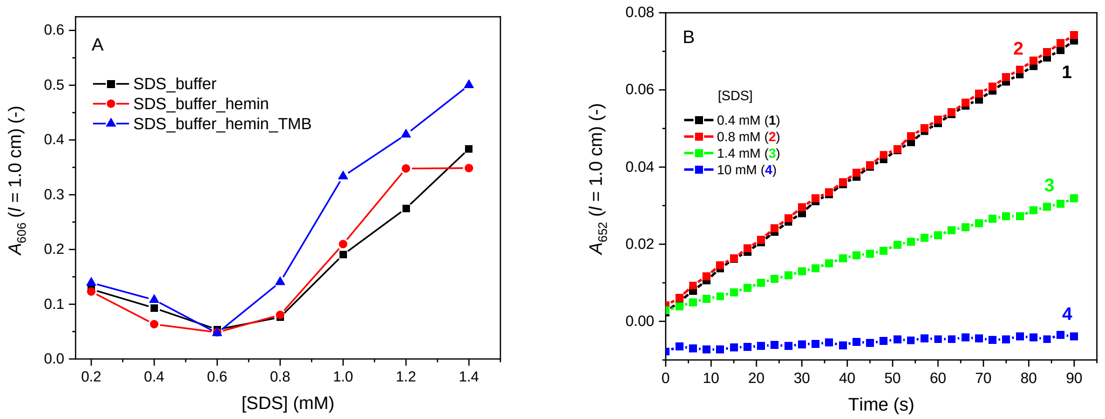

Figure S7. (A) Determination of the cmc of SDS with pinacyanol chloride (5 µM) in the presence of 100 mM sodium phosphate buffer solution, pH = 7.2 at T = 25 °C, either (i) in the absence of hemin or TMB (■), (ii) in the presence of 250 nM hemin (●), or (iii) in the presence of 250 nM hemin and 0.3 mM TMB (▲). The SDS concentration was varied between 0.2 and 1.4 mM (in steps of 0.2 mM). *Result:* In the presence of hemin and TMB, the cmc of SDS was about 0.6 − 0.8 mM. **(B)** Peroxidase-like activity of hemin against TMB as reducing substrate in the presence of SDS in phosphate buffer solution at pH = 7.2. *Reaction conditions:* [phosphate] = 100 mM; [hemin] = 250 nM; [TMB] = 0.3 mM; [H_2O_2] = 0.3 mM; RT. The absorption spectrum of the reaction mixture was recorded every 3 s for a total of 90 s. A_{562} is plotted against reaction time as a measure of the formation of the CTC. Using ε_{652} (CTC) = 39,000 $M^{-1}cm^{-1}$,[S5] the initial rate of CTC formation, v_{in}(CTC), in the presence of 0.4 or 0.8 mM SDS was calculated to v_{in}(CTC) ≈ 20 nM s^{-1}. *Results:* The initial rate of CTC formation was higher at [SDS] = 0.4 or 0.8 mM than at [SDS] = 1.4 or 10 mM. In the presence of 100 mM sodium phosphate solution (pH = 7.2) at 0.4 or 0.8 mM SDS, v_{in}(CTC) was ≈15 times lower than in the presence of 100 mM HEPES buffer solution (pH = 7.2) at the optimal SDS concentration of 2.0 mM (≈20 nM s^{-1} *vs.* ≈300 nM s^{-1}, see **Figure 6).**

5. Effect of SDS on the Peroxidase-like Activity of Hemin Using TMB as Reducing Substrate

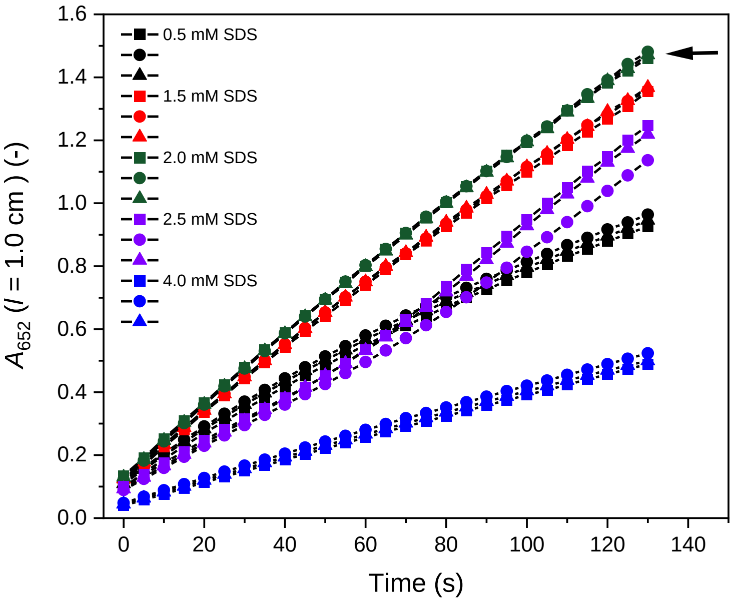

Figure S8. Effect of SDS on the initial rate of CTC formation from TMB as reducing substrate, monitored by recording A_{652} as a function of reaction time ($N = 3$). *Reaction conditions:* [HEPES] = 100 mM; pH = 7.2; [SDS] = 0.5, 1.5, 2.0, 2.5, or 4.0 mM; [hemin] = 250 nM; [TMB] = 0.3 mM; [H_2O_2] = 0.3 mM; RT. *Results:* For the chosen SDS concentrations, the fastest initial increase in A_{652} was for 2.0 mM SDS, correlating with the determined cmc of SDS in the presence of 100 mM HEPES (1.9 − 2.0 mM, see **Figure S4**). The TMB conversion after 130 s (arrow) was ≈38 µM, *i.e.,* ≈12.5%. The reaction was low at 4.0 mM SDS. At 2.5 mM SDS, the A_{652} vs. time curve showed a kink after ≈70 s, the CTC formation becoming faster. There was no indication of a rapid leveling-off of the CTC formation as observed in the absence of SDS, please compare with **Figure 4**.

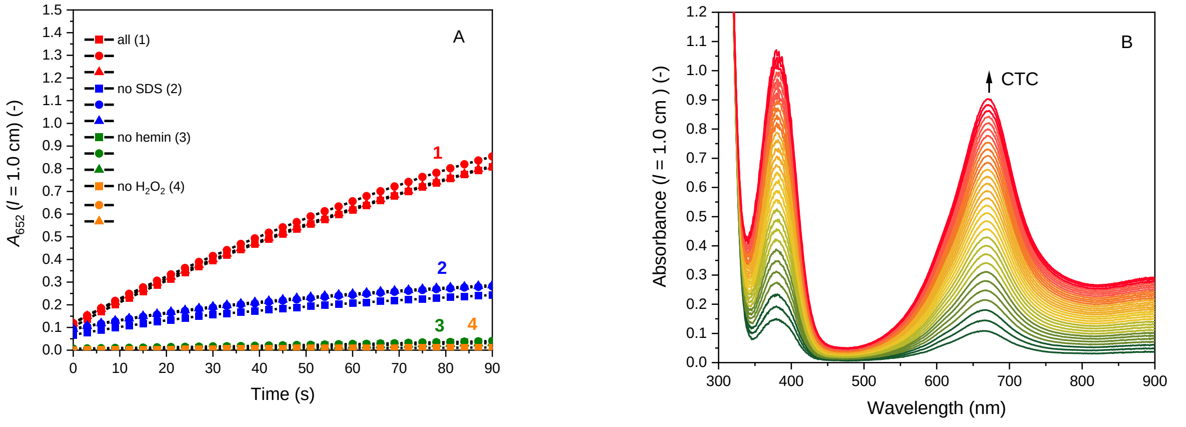

Figure S9. Comparison of the effect of SDS (2.0 mM) on the progress of the peroxidase-like activity of hemin measured with TMB as reducing substrate, and control measurements in the absence of either hemin or H_2O_2. **(A)** Initial formation of the CTC, determined by recording A_{652} vs. time. Reaction conditions for "all": [HEPES] = 100 mM; pH = 7.2; [SDS] = 2.0 mM; [hemin] = 250 nM; [TMB] = 0.3 mM; [H_2O_2] = 0.3 mM; RT; N = 3. For the other conditions, either SDS, hemin or H_2O_2 was omitted. *Results:* Without hemin or H_2O_2, the reaction did not take place. Without SDS, the reaction was slow and leveled-off (see also **Figure 4**). In the presence of 2.0 mM SDS, the reaction was faster than in the absence of SDS and did not level-off within the time of observation. **(B)** Time dependent changes of the absorption spectrum of the reaction mixture for the conditions "all" in **(A)**. *Results:* The reaction product formed in the presence of SDS – at least during the initial phase of the reaction – has always the same typical spectrum of the CTC. Compared to the reactions run in the absence of SDS, the intensity of the band centered around λ = 900 nm was much lower; compare the spectrum shown in **(B)** with the spectrum for 100 mM HEPES conditions shown in **Figure S1**. No indication for the formation of a second-electron oxidation product. The control experiments confirm the expected absence of significant reaction if either hemin or H_2O_2 was omitted.

6. Effect of SDS on the Stability of Aqueous Hemin Solutions Kept Inside Polystyrene Cuvettes

On the basis of previous findings about the adsorption of hemin from aqueous solution onto plasticware,[S6] we investigated whether the presence of SDS has an influence on this adsorption process, and whether the SDS concentration dependence of the peroxidase-like activity of hemin measured with TMB (**Figure 6**) could be explained by differences in the rate and/or extent of hemin adsorption to the polystyrene cuvettes used for the activity measurements (see section 2.4). For this, aqueous 100 mM HEPES buffer solutions of pH = 7.2 containing hemin (250 nM) and different amounts of SDS (up to 3.0 mM) were added to polystyrene cuvettes, and the UV−vis absorption spectra and the activity of these solutions were measured from time to time during an incubation at RT of up to 2 h, see **Figure S10**. In all cases, the Soret band intensity of hemin at λ = 396 nm, A_{396}, decreased with storage time at similar rate (**Figure S10A**), and in all cases, v_{in}(CTC) decreased with storage time with similar rate (**Figure S10B**). Therefore, we conclude that hemin adsorption on the polystyrene cuvette wall was not the reason for the variation of v_{in}(CTC) with SDS concentration shown in **Figure 6**.

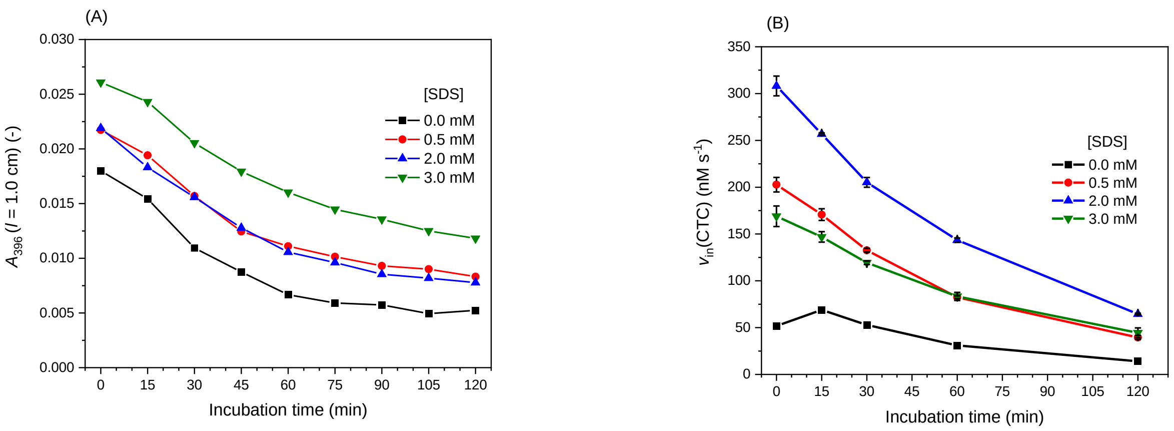

Figure S10. Storage stability of hemin (250 nM) in aqueous solution in the presence of SDS, kept inside disposable polystyrene cuvettes at RT for up to 120 min. [HEPES] = 100 mM; pH = 7.2; [SDS] = 0.0, 0.5, 2.0, or 3.0 mM. **(A)** Change in the Soret band intensity of hemin at λ = 396 nm, A_{396}, measured every 15 min. **(B)** Change in the initial rate of CTC formation, v_{in}(CTC), measured with TMB as reducing substrate after 0, 15, 30, or 120 min of incubation; N = 3. TMB (0.3 mM) and H_2O_2 (0.3 mM) were added after the indicated time, and the initial change in A_{652} was measured and then converted into units of nM CTC formed per second (nM s^{-1}) by using ε_{652} (CTC) = 39,000 $M^{-1}cm^{-1}$.[S5] *Comment:* Based on literature,[S6] the decrease in A_{396} and in v_{in} with storage time can be ascribed to the adsorption of hemin onto the inner polystyrene wall of the cuvettes used.

7. Effect of L-His on the Peroxidase-like Activity of Hemin in the Presence and Absence of SDS Using TMB as Reducing Substrate

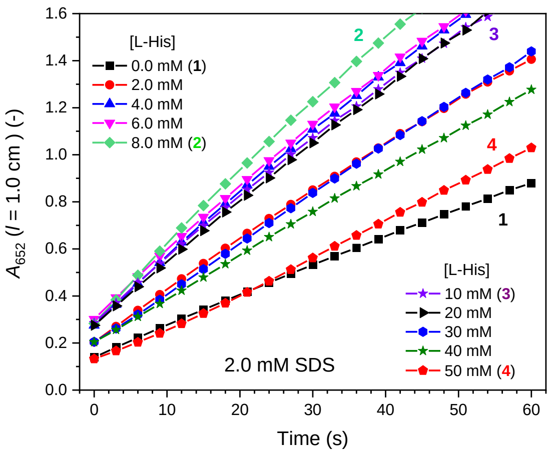

Figure S11. Effect of L-His on the initial rate of CTC formation from TMB as reducing substrate with hemin as catalyst *in the presence of 2.0 mM SDS*, monitored by recording A_{652} as a function of reaction time. *Reaction conditions:* [HEPES] = 100 mM; pH = 7.2; [SDS] = 2.0 mM; [hemin] = 250 nM; [L-His] = 0.0, 2.0, 4.0, 6.0, 8.0, 10, 20, 30, 40 or 50 mM; [TMB] = 0.3 mM; [H_2O_2] = 0.3 mM; RT; N = 3 (for the sake of clarity, only one measurement is shown for each L-His concentration). Data from which **Figure 9A** was prepared. *Results:* A_{652} increased continuously during the first 60 seconds, the reaction for [L-His] = 8.0 mM being fastest.

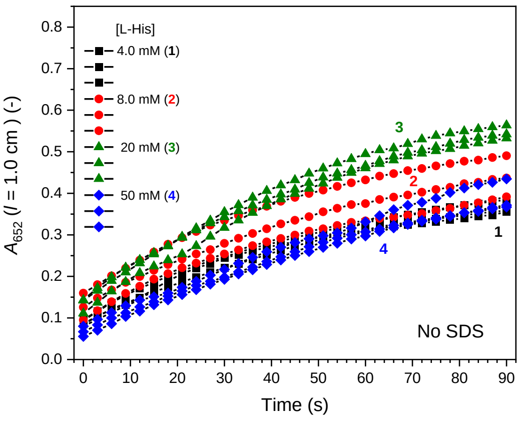

Figure S12. Effect of L-His on the initial rate of CTC formation from TMB as reducing substrate with hemin as catalyst *in the absence of SDS*, monitored by recording A_{652} as a function of reaction time. *Reaction conditions:* [HEPES] = 100 mM; pH = 7.2; [hemin] = 250 nM; [L-His] = 4.0, 8.0, 20, or 50 mM; [TMB] = 0.3 mM; [H_2O_2] = 0.3 mM; RT; N = 3. *Results:* A_{652} increased with time with a trend to level off. The highest initial rate of CTC formation within this set of experiments was ≈ 170 nM s^{-1} (in the presence of 20 mM L-His), which is significantly lower than in the case of the optimal system in the presence of 2.0 mM SDS (8.0 mM L-His: v_{in}(CTC) ≈ 850 nM s^{-1}), see **Figure 9a** and **Figure S11**.

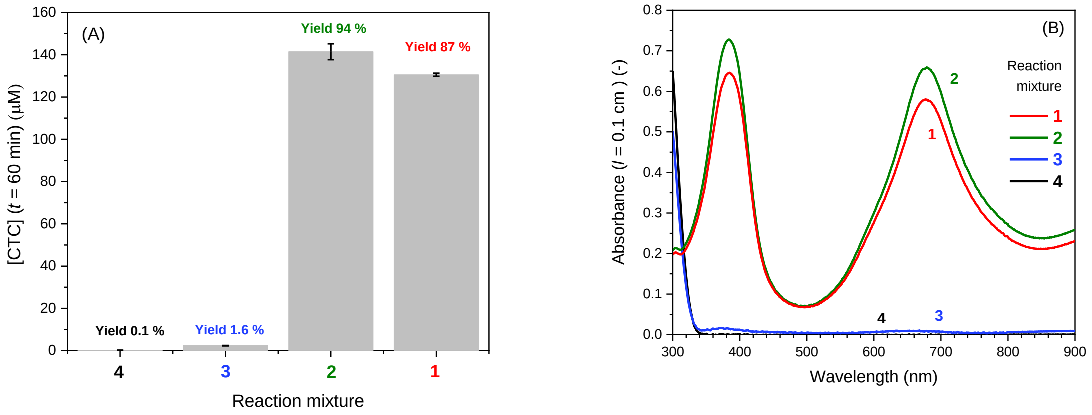

Figure S13. **(A)** Determination of the reaction yield after a reaction time of 60 min at RT for the hemin-catalyzed oxidation of TMB (0.3 mM) in the presence of SDS (2.0 mM) and L-His (8.0 mM) using 250 nM hemin and 0.3 mM (**1**), and comparison with the reaction yield obtained for the reaction run either without L-His (**2**), without L-His and SDS (**3**), or without L-His, SDS, and H_2O_2 (**4**). *Reaction conditions:* (**1**): [HEPES] = 100 mM; pH = 7.2; [SDS] = 2.0 mM; [hemin] = 250 nM; [L-His] = 8.0 mM; [TMB] = 0.3 mM; [H_2O_2] = 0.3 mM, $N = 3$. (**2**) [HEPES] = 100 mM; pH = 7.2; [SDS] = 2.0 mM; [hemin] = 250 nM; [TMB] = 0.3 mM; [H_2O_2] = 0.3 mM, $N = 3$. (**3**) [HEPES] = 100 mM, pH = 7.2, [hemin] = 250 nM; [TMB] = 0.3 mM; [H_2O_2] = 0.3 mM, $N = 3$. (**4**) [HEPES] = 100 mM, pH = 7.2, [hemin] = 250 nM; [TMB] = 0.3 mM; $N = 3$. **(B)** Comparison of the UV−vis absorption spectra of the reaction mixtures (**1**) – (**4**) after a reaction time of 60 min. The CTC yields were calculated by using ε_{652} (CTC) = 39,000 $M^{-1}cm^{-1}$.[S5] The absorption spectra shown were measured by withdrawing volumes of 350 µL from the reaction mixtures and placing the aliquots inside a quartz cuvette of pathlength 0.1 cm, *i.e.*, neither dilution nor work-up of the reaction mixtures was required. *Results:* The yields of the charge transfer complex (CTC) after 60 min were 87 % for (**1**), 94 % for (**2**), 1.6 % (for **3**), and 0.1 % for (**4**).

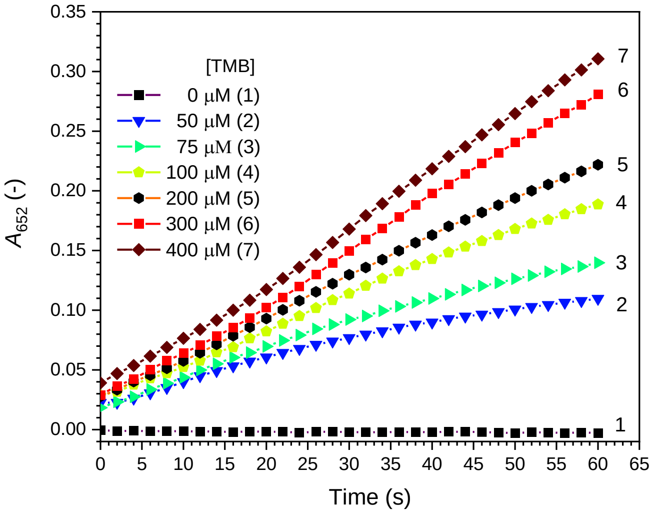

Figure S14. TMB concentration dependence of the initial rate of CTC formation from TMB as reducing substrate and hemin as a catalyst *in the presence of SDS* and L-His by recording A_{652} as a function of reaction time. Due to the non-linear initial increase of A_{652} with time, v_{in}(CTC) was determined from the changes occurring after 25 s using ε_{652} (CTC) = 39,000 $M^{-1}cm^{-1}$.[S5]. *Reaction conditions:* [HEPES] = 100 mM; pH = 7.2; [SDS] = 2.0 mM; [hemin] = 5 nM; [L-His] = 8.0 mM; [TMB] = 0, 50, 75, 100, 200, 300, and 400 μM; [H_2O_2] = 1.0 mM; N = 3; RT. The UV−vis absorption spectra of the reaction mixtures were recorded every 2 s for 60 s. For the sake of clarity, only one measurement is shown for each TMB concentration.

A plot of v_{in}(CTC) vs. [TMB] is shown in **Figure 12** with a fit of the experimental data to the Michaelis-Menten equation (using OriginPro, Version 2021; OriginLab Corporation, Northampton, MA, USA). The fit yielded $v_{max,app}$ = 193 ± 16 nM s^{-1}, *i.e.*, $k_{cat,app} = v_{max,app}$ / [Hemin] = 39 ± 3 s^{-1}, and $K_{M,app}$ = 253 ± 37 μM. *Comment:* The non-linear change of A_{652} with time indicates a complex kinetic behavior, possibly due to influences of the formed product on the reaction.

Model	Michaelis Menten
Equation	y = Vmax * x / (Km + x)
Plot	D
Vmax	193.3 ± 16.04
Km	252.6 ± 37.08
Reduced Chi-Sqr	4.64558
R-Square (COD)	0.99929
Adj. R-Square	0.99915

8. Determination of $K_M(H_2O_2)$ and $k_{cat}(H_2O_2)$ of HRP Measured with TMB as Reducing Substrate

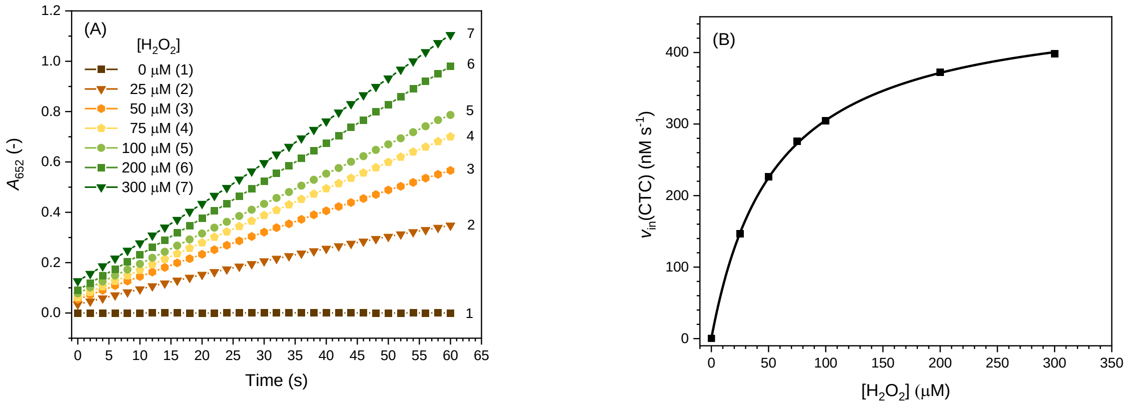

Figure S15. H_2O_2 concentration dependence of the initial rate of CTC formation from TMB as reducing substrate and HRPC as a catalyst in HEPES buffer solution of pH = 7.2. **(A)** A_{652} *vs.* time. **(B)** v_{in}(CTC) *vs.* [H_2O_2]; v_{in}(CTC) being determined form the first 30 s of reaction by taking into account ε_{652} (CTC) = 39,000 $M^{-1}cm^{-1}$.[S5] *Reaction conditions:* [HEPES] = 100 mM; pH = 7.2; [HRPC] = 5 nM; [TMB] = 300 μM; [H_2O_2] = 0, 25, 50, 75, 100, 200, and 300 μM; *N* = 3; RT. The UV−vis absorption spectra of the reaction mixtures were recorded every 2 s for 60 s.

Model	Michaelis Menten
Equation	y = Vmax * x / (Km + x)
Plot	d[CTC]/dt
Vmax	474.6 ± 2.35
Km	55.5 ± 0.69
Reduced Chi-Sqr	0.58984
R-Square (COD)	0.99996
Adj. R-Square	0.99995

The fit of the experimental data to the Michaelis-Menten equation (using OriginPro, Version 2021; OriginLab Corporation, Northampton, MA, USA) yielded: v_{max} = 474.6 ± 2.4 nM s^{-1}, *i.e.*, $k_{cat} = v_{max}$ / [HRPC] = 94.9 ± 0.4 s^{-1}, and K_M = 56 ± 1 μM.

9. Effect of H_2O_2 on the Peroxidase-like Activity of Hemin/SDS/L-His Using TMB as Reducing Substrate

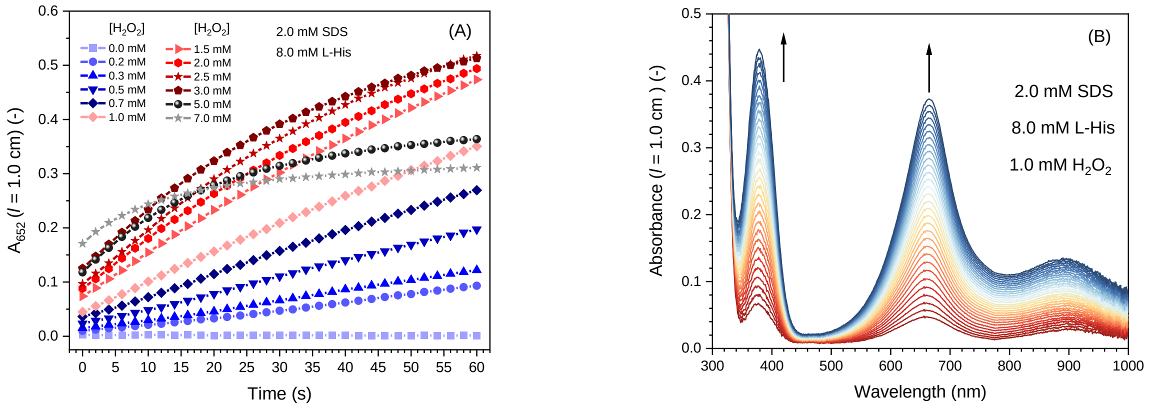

Figure S16. (A) Effect of H_2O_2 on the initial rate of CTC formation from TMB (0.3 mM) for the hemin/SDS/L-His system in 100 mM HEPES buffer solution (pH = 7.2) containing 5 nM hemin, 2.0 mM SDS and 8.0 mM L-His. A_{652} is plotted as function of reaction time. The reaction was run at RT with [H_2O_2] = 0.0, 0.2, 0.3, 0.5, 0.7, 1.0, 1.5, 2.0, 2.5, 3.0, 5.0, and 7.0 mM; N = 3. These are the data from which **Figure 11** was prepared. For the sake of clarity, for each H_2O_2 concentration used only one measurement is shown for a reaction time of 60 s. The reaction mixtures were prepared by mixing appropriate stock solutions of the different compounds in the following order: 1. HEPES; 2. SDS; 3. Hemin; 4. L-His; 5. TMB; 6. H_2O_2. **(B)** Illustration of the change of the UV-vis absorption spectrum of one of the reaction mixtures run with 1.0 mM H_2O_2. The spectrum was recorded every 2 s for a total of 60 s.

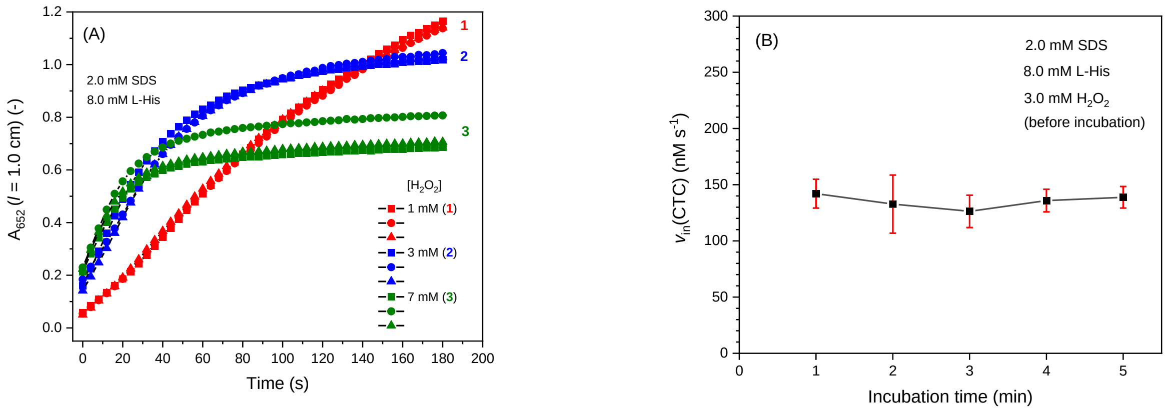

Figure S17. Peroxidase-like activity of hemin (5 nM) in 100 mM HEPES buffer solution (pH = 7.2) in the presence of 2.0 mM SDS, and 8.0 mM L-His, measured with TMB (0.3 mM) as reducing substrate and H_2O_2 as oxidizing substrate at RT. **(A)** For [H_2O_2] = 1.0, 3.0, and 7.0 the time progress is shown for up to 3 min. The reaction mixtures were prepared by mixing appropriate stock solutions of the different compounds in the following order: 1. HEPES; 2. SDS; 3. Hemin; 4. L-His; 5. TMB; 6. H_2O_2. *Results:* For high concentrations of H_2O_2, the increase of A_{652} with time was not linear. A_{652} leveled-off the earlier the higher the H_2O_2 concentration was. **(B)** Preincubation experiments with 3.0 mM H_2O_2. Except for TMB, all components were first incubated for either 1, 2, 3, 4, or 5 min at RT before a stock solution of TMB in DMSO was added to start the reaction (N = 3, except for 2 min incubation: N = 2). The progress of the reaction was followed for 60 s by recording the entire UV−vis absorption spectrum of the reaction mixture every 2 s and monitoring A_{652} from which v_{in}(CTC) was calculated using ε_{652} (CTC) = 39,000 $M^{-1}cm^{-1}$.[S5] *Result:* Inactivation of hemin by hydrogen peroxide occurred in a period of less than one minute, and the degree of hemin inactivation remained approximately the same for all incubation times tested.

10. Effect of the Hemin Concentration on the Peroxidase-like Activity of Hemin/SDS/L-His Using TMB as Reducing Substrate

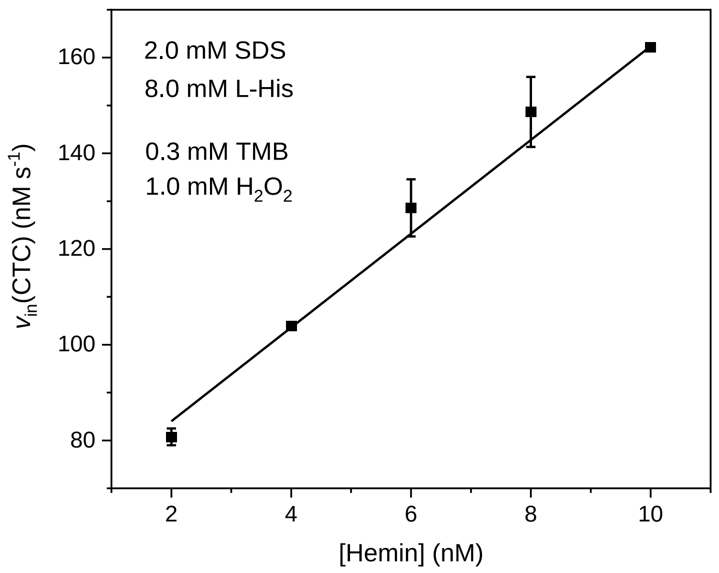

Figure S18. Peroxidase-like activity of hemin in 100 mM HEPES buffer solution (pH = 7.2) in the presence of 2.0 mM SDS, and 8.0 mM L-His, measured with TMB (0.3 mM) as reducing substrate and H_2O_2 (1.0 mM) as oxidizing substrate at RT. The concentration of hemin was varied: 2.0, 4.0, 6.0, 8.0, or 10.0 nM, $N = 3$. The reaction mixtures were prepared by mixing appropriate stock solutions of the different compounds in the following order: 1. HEPES; 2. SDS; 3. Hemin; 4. L-His; 5. TMB; 6. H_2O_2. The initial rate of CTC formation, v_{in}(CTC), is plotted against the hemin concentration. *Result:* Linear dependence of v_{in}(CTC) on the hemin concentration between [hemin] = 2 and 10 nM.

11. Effect of the Presence of SDBS, CTAB, or Triton X-100 on the Peroxidase-like Activity of Hemin in HEPES Buffer Solution at pH = 7.2, Measured with TMB as Reducing Substrate

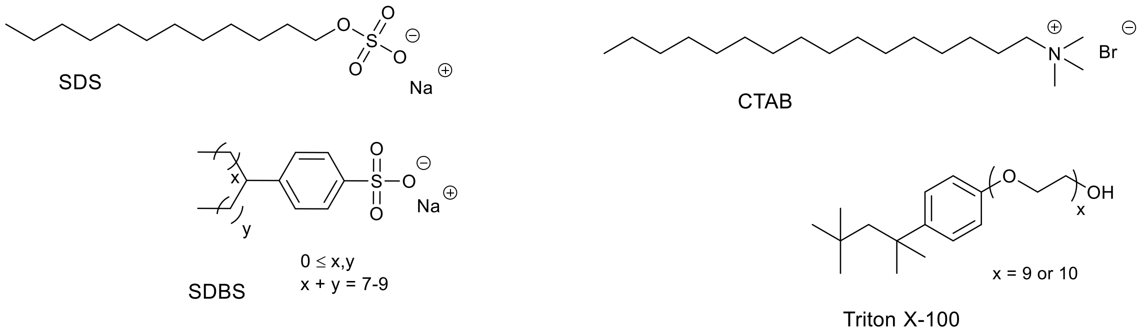

Figure S19. Chemical structures of the surfactants used in this work SDS, SDBS,[S7,S8] CTAB, and Triton X-100.

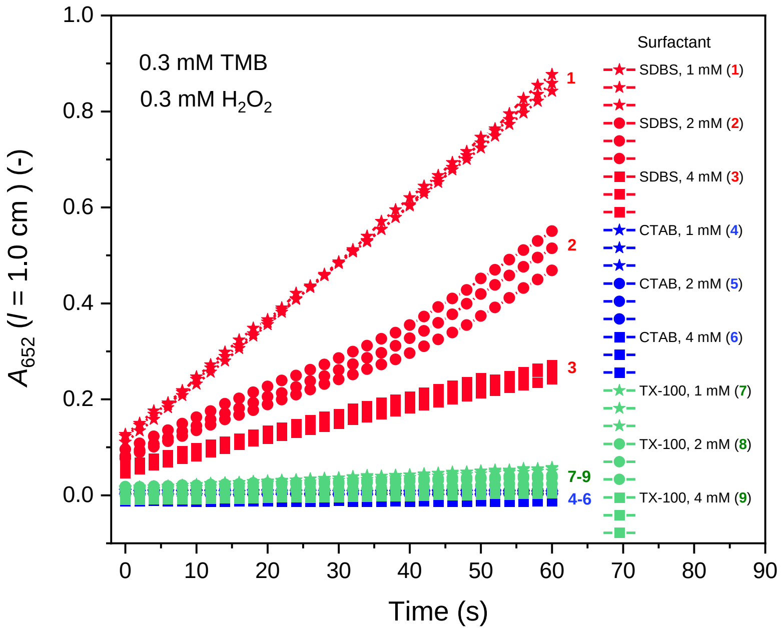

Figure S20. Peroxidase-like activity of hemin against TMB as reducing substrate in HEPES buffer solution at pH = 7.2 in the presence of either *SDBS*, *CTAB*, or *Triton X-100*. A_{652} is plotted as a function of time (up to 60 s) as indication of the formation of the CTC. The UV−vis absorption spectra of the reaction mixtures were measured every 3 s (for SDBS, see **Figure S21**). *Reaction conditions:* [HEPES] = 100 mM; pH = 7.2; [SDBS] or [CTAB] or [Triton X-100] = 1.0, 2.0, or 4.0 mM; [hemin] = 250 nM; [TMB] = 0.3 mM; [H_2O_2] = 0.3 mM; N = 3, RT.

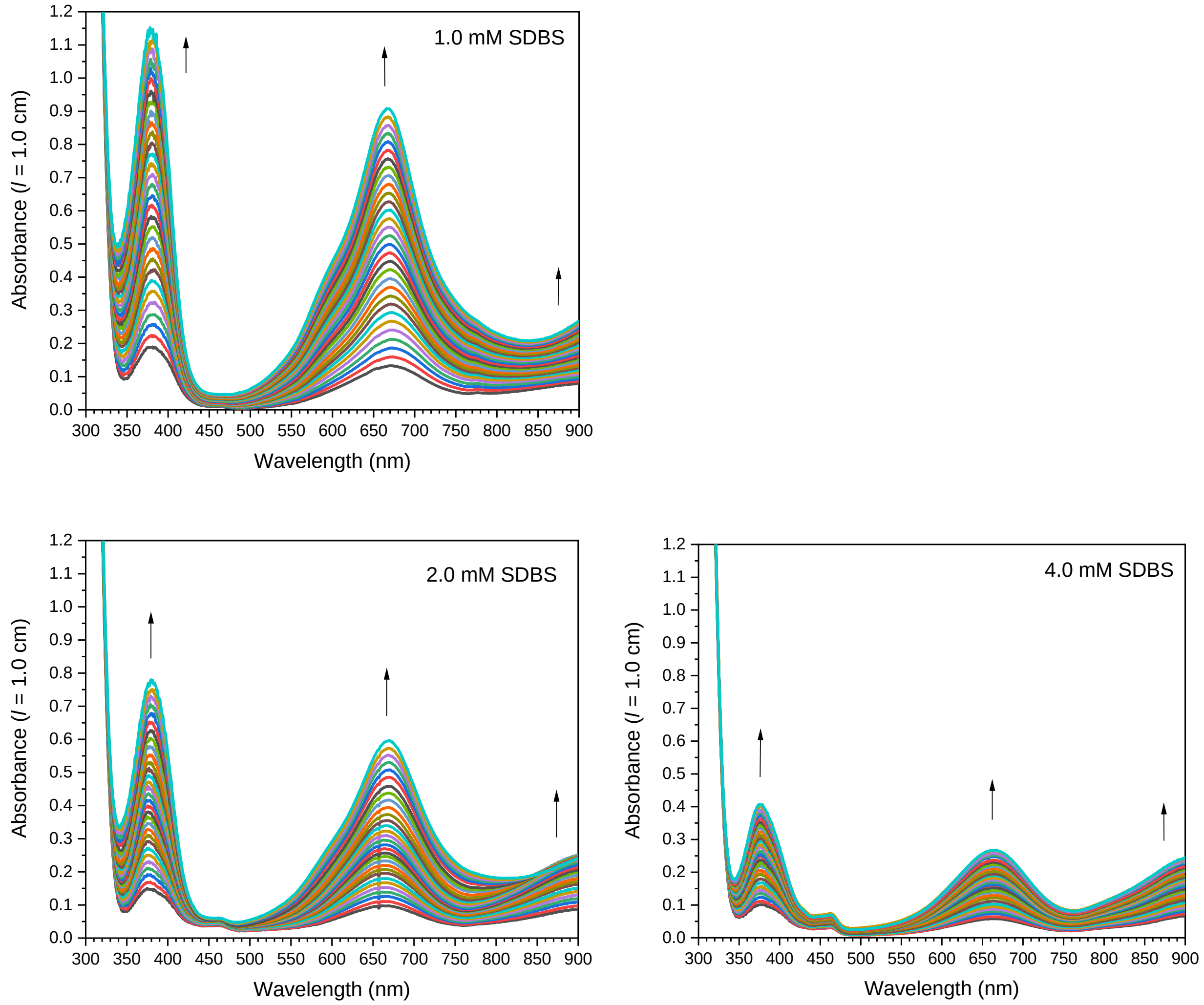

Figure S21. Hemin-catalyzed oxidation of TMB in 100 mM HEPES buffer solution (pH = 7.2) containing SDBS. Examples of the development of the UV−vis absorption spectra of the reaction mixtures over time for different concentrations of SDBS. *Reaction conditions:* [HEPES] = 100 mM; pH = 7.2; [SDBS] = 1.0, 2.0, or 4.0 mM; [hemin] = 250 nM; [TMB] = 0.3 mM; [H_2O_2] = 0.3 mM; RT. The UV−vis absorption spectra were measured every 3 s for 60 s. Results: For all three SDBS concentrations used, bonds cantered around 370, 650, and 900 nm developed with time, in agreement with the formation of the CTC, see **Figure 2**. Depending on the SDBS concentration, however, there were differences in (i) the rate at which the intensity of the three bands increased, (ii) the ratio of A_{652} to A_{900}, (iii) the situation at $\lambda \approx 850$ nm in the case of 2.0 mM SDBS (reproducibly observed for all three measurements at 2.0 mM SDBS, with a kink in the A_{652} vs. time line, see **Figure S20**), and (iv) the appearance of a small peak at $\lambda \approx 460$ nm.

12. Peroxidase-like Activity of Hemin in HEPES Buffer Solution (pH = 7.2) in the Presence of SDS Against $ABTS^{2-}$, Amplex Red, or $DCFH_2$ as Reducing Substrates

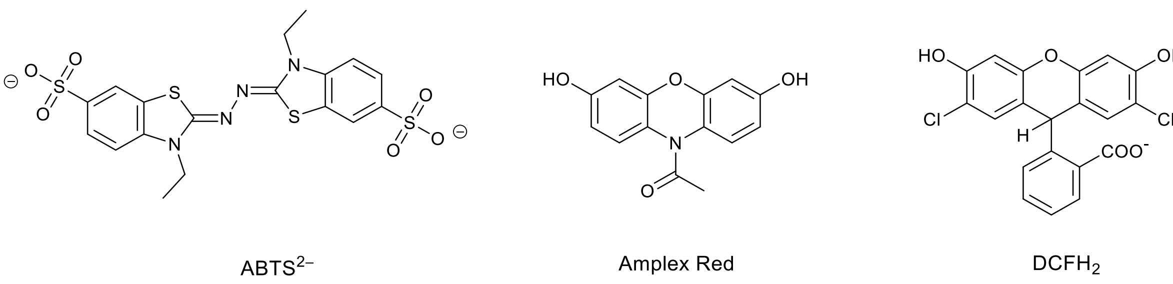

Figure S22. Chemical structures of $ABTS^{2-}$, Amplex Red, and $DCFH_2$.[S9]

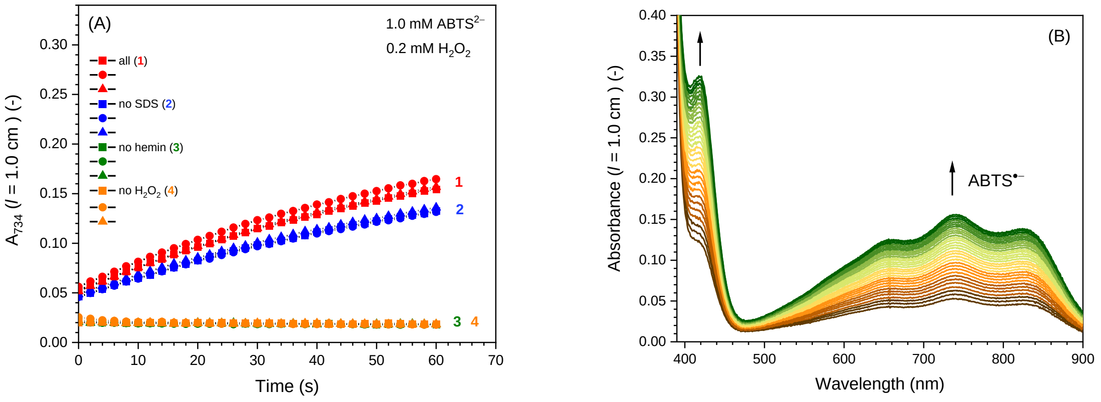

Figure S23. Peroxidase-like activity of hemin in 100 mM HEPES buffer solution (pH = 7.2) in the presence of 2.0 mM SDS against *$ABTS^{2-}$* as reducing substrate. *Reaction conditions ("all"):* [HEPES] = 100 mM; pH = 7.2; [SDS] = 2.0 mM; [hemin] = 250 nM; [$ABTS^{2-}$] = 1.0 mM; [H_2O_2] = 0.2 mM; $N = 3$, RT. In a reference measurement, SDS was omitted ("no SDS"). In two control measurements either hemin ("no hemin") or H_2O_2 ("no H_2O_2") was omitted. **(A)** A_{734} is plotted against reaction time, indicative for the formation of $ABTS^{\bullet-}$; ε_{734} ($ABTS^{\bullet-}$) = 18,200 $M^{-1}cm^{-1}$ (Childs and Bardsley, 1975).[S10] **(B)** Time-dependent change of the UV−vis absorption spectrum of the reaction mixture "all", demonstrating continuous $ABTS^{\bullet-}$ product formation during the initial stage of the reaction, see also Cvjetan and Walde (2023).[S11]

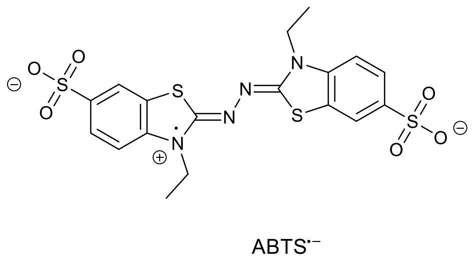

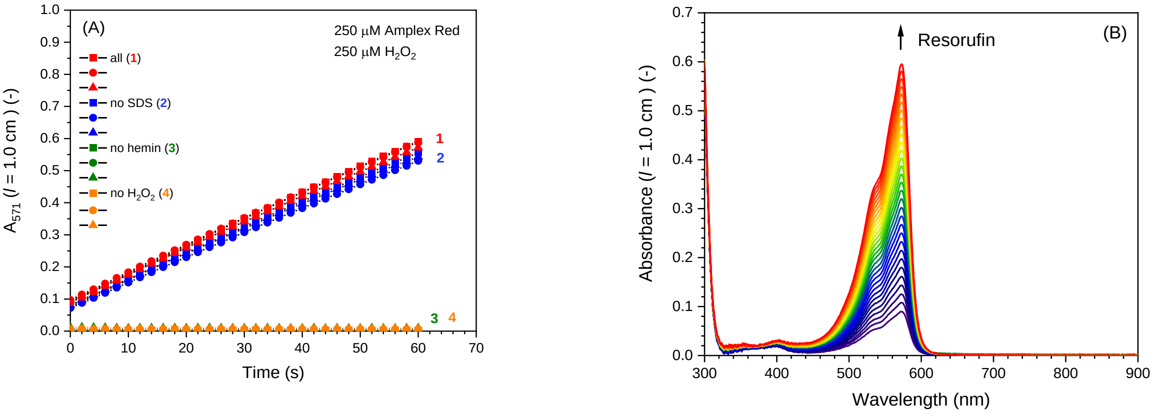

Figure S24. Peroxidase-like activity of hemin in 100 mM HEPES buffer solution (pH = 7.2) in the presence of 2.0 mM SDS against *Amplex Red* as reducing substrate. *Reaction conditions ("all"):* [HEPES] = 100 mM; pH = 7.2; [SDS] = 2.0 mM; [hemin] = 250 nM; [Amplex Red] = 250 µM; [H_2O_2] = 250 µM; N = 3, RT. In a reference measurement, SDS was omitted ("no SDS"). In two control measurements either hemin ("no hemin") or H_2O_2 ("no H_2O_2") was omitted. **(A)** A_{571} is plotted against reaction time, indicative for the formation of resorufin; ε_{570} (resorufin) ≈ 57,000 $M^{-1}cm^{-1}$ (Oja et al., 2014)[S12] **(B)** Time-dependent change of the UV−vis absorption spectrum of the reaction mixture "all", demonstrating continuous resorufin product formation during the initial stage of the reaction, see also Cvjetan and Walde (2023).[S11]

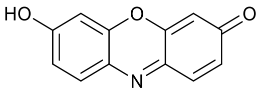

Resorufin

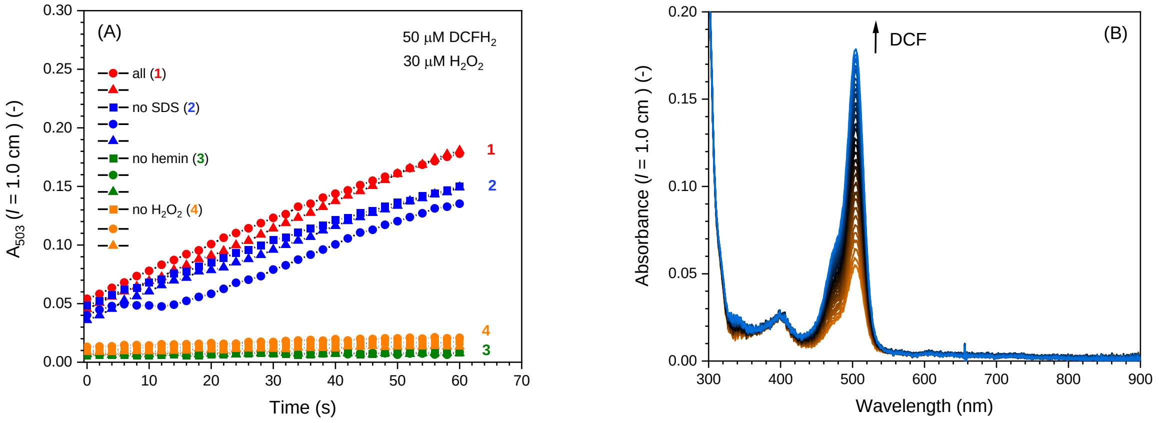

Figure S25. Peroxidase-like activity of hemin in 100 mM HEPES buffer solution (pH = 7.2) in the presence of 2.0 mM SDS against *DCFH*$_2$ *(= 2'7'-dichlorodihydrofluorescein)* as reducing substrate. *Reaction conditions ("all"):* [HEPES] = 100 mM; pH = 7.2; [SDS] = 2.0 mM; [hemin] = 250 nM; [DCFH$_2$] = 50 µM; [H_2O_2] = 30 µM; $N = 3$, RT. In a reference measurement, SDS was omitted ("no SDS"). In two control measurements either hemin ("no hemin") or H_2O_2 ("no H_2O_2") was omitted. **(A)** A_{503} is plotted against reaction time, indicative for the formation of DCF (2',7'-dichlorofluorescein; ε_{503} (DCF) = 101,900 $M^{-1}cm^{-1}$ (Ghéczy *et al.*, 2020)[S9] **(B)** Time-dependent change of the UV−vis absorption spectrum of the reaction mixture "all", demonstrating continuous DCF product formation during the initial stage of the reaction, see Ghéczy et al. (2020)[S9] for details of the reaction.

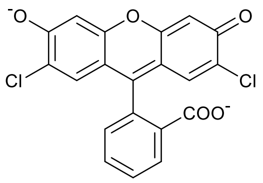

DCF

13. Experiments with Vesicle Dispersions Prepared from SDS and Dodecanol in HEPES Buffer Solution (pH = 7.2)

Vesicle Preparation

Aqueous dispersions of vesicles composed of SDS and dodecanol were prepared at RT by the procedure reported by Hargreaves and Deamer (1978)[S13] with some modifications, followed by polycarbonate membrane extrusion using a LiposoFast device.[S14] As aqueous medium 100 mM HEPES buffer solution (pH = 7.2) was used and a total SDS + dodecanol concentration of 50 mM of which 70 mol% was dodecanol ([SDS] = 15 mM; [dodecanol] = 35 mM). 70 mol% dodecanol was chosen since at this dodecanol content, the obtained vesicle dispersion was the most stable one among the ones tested (40, 50, 60, or 70 mol% dodecanol). The vesicle dispersion was prepared in a transparent 5 mL Eppendorf tube by first dissolving the desired amount of SDS in 100 mM HEPES buffer solution (pH = 7.2). Afterwards, the solution was warmed up by placing the tube in a water bath of 50 °C. Melted dodecanol was added dropwise to the SDS solution by using a micropipette. The mixture was vortexed for ≈1 min at intermediate speed (using a Vortex Genie 2 instrument), submerged for 1 min in a water batch set to 50 °C and then vortexed again for ≈1 min at the same intermediate speed. The obtained dispersion was extruded 11 times through 100 nm Nucleopore polycarbonate membranes using a LiposoFast device (from Avestin).[S14] The obtained vesicle dispersion was kept at RT and used within 1 week. Dispersions of a desired concentration of SDS and dodecanol for the activity measurements were obtained through dilution with 100 mM HEPES buffer solution (pH = 7.2) just before carrying out the measurements.

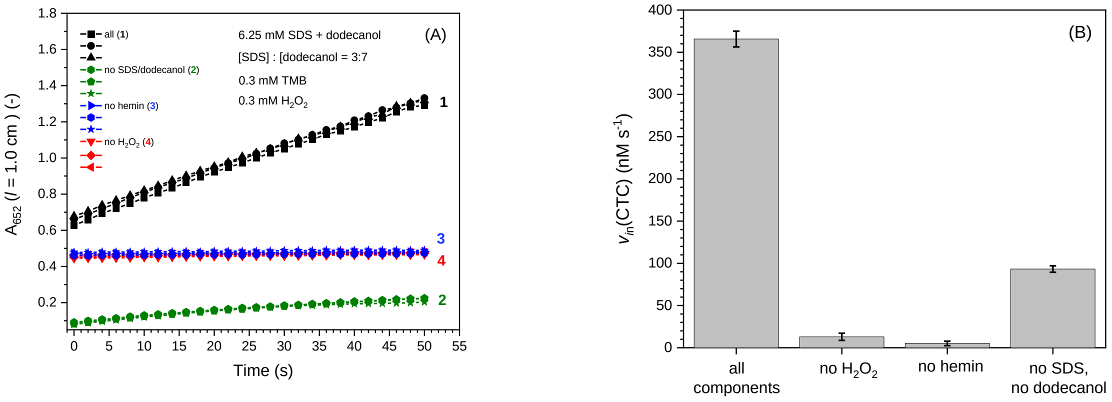

Figure S26. Peroxidase-like activity of hemin in 100 mM HEPES buffer solution (pH = 7.2) in the presence of SDS:dodecanol (3:7, mol ratio) vesicles against TMB as reducing substrate. *Reaction conditions ("all"):* [HEPES] = 100 mM; pH = 7.2; [SDS] + [dodecanol] = 6.25 mM ([SDS]:[dodecanol] = 3:7); [hemin] = 250 nM; [TMB] = 0.3 mM; [H_2O_2] = 0.3 mM; RT; $N = 3$. In a reference measurement, SDS and dodecanol were omitted ("no SDS/dodecanol"). In two control measurements either hemin ("no hemin") or H_2O_2 ("no H_2O_2") was omitted. **(A)** Changes in A_{652} with reaction time as indication of CTC formation. **(B)** Comparison of the measured initial rates of CTC formation for the different reaction mixtures, determined by considering as molar absorption coefficient ε_{652} (CTC) = 39,000 $M^{-1}cm^{-1}$.[S5]

Batch-to-Batch Variation of the Peroxidase-like Activity of Hemin in SDS:Dodecanol (3:7) Vesicle Dispersions

The SDS/dodecanol vesicle dispersions prepared by polycarbonate extrusion are only kinetically stable and not thermodynamically. Therefore, checking the reproducibility of any type of experiment with vesicular dispersions is important. We measured the peroxidase-like activity of hemin under the "optimal conditions" towards TMB as reducing substrate by using five different batches of prepared SDS:dodecanol (3:7) vesicles. The determined initial rates of CTC formation for the five reaction mixtures are given in **Figure S27**. The values of v_{in}(CTC) varied between ≈300 and ≈480 nM s^{-1}; see also **Figure S26A** (entry "all"). As a conclusion, it is recommended to use for one comparative set of measurements always the same batch of vesicles. This was done for the data shown in **Figure S28** (effect of added L-His on the activity of hemin in the SDS/dodecanol vesicle dispersion).

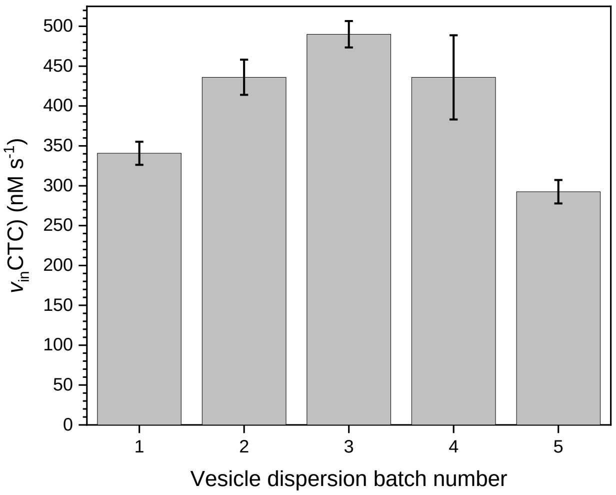

Figure S27. Reproducibility check for the peroxidase-like activity of hemin in a SDS/dodecanol vesicles dispersion towards TMB as a reducing substrate. *Reaction conditions:* [HEPES] = 100 mM; pH = 7.2; [SDS] + [dodecanol] = 6.25 mM ([SDS]:[dodecanol] = 3:7); [hemin] = 250 nM; [TMB] = 0.3 mM; [H_2O_2] = 0.3 mM; RT; N = 3. Five batches of vesicle dispersion were prepared in the same way. The initial rate of CTC formation, v_{in}(CTC), was determined from A_{652} *vs.* time plots of reactions run for 50 s, using considering ε_{652}(CTC) = 39,000 $M^{-1}cm^{-1}$.[S5]

Effect of L-His on the Peroxidase-like Activity of Hemin in SDS:Dodecanol (3:7) vesicle Dispersions

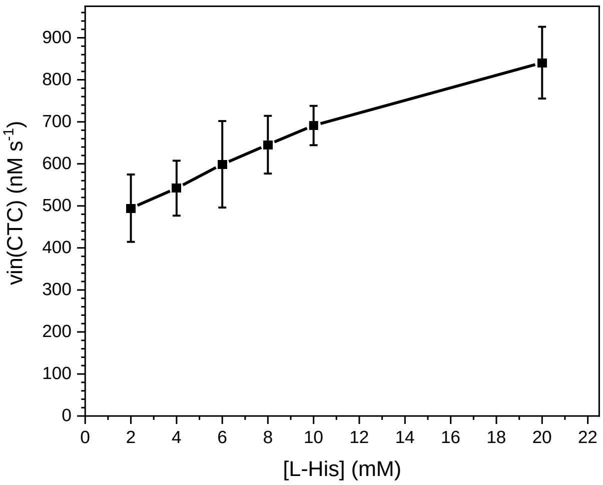

Figure S28. Effect of L-His on the peroxidase-like activity of hemin in a SDS/dodecanol vesicles dispersion towards TMB as a reducing substrate. *Reaction conditions:* [HEPES] = 100 mM; pH = 7.2; [SDS] + [dodecanol] = 6.25 mM ([SDS]:[dodecanol] = 3:7); [hemin] = 250 nM; [L-His] = 2.0, 4.0, 8.0, 10, or 20 mM; [TMB] = 0.3 mM; [H_2O_2] = 0.3 mM; RT; $N = 3$. The initial rate of CTC formation, v_{in}(CTC), was determined from A_{652} *vs.* time plots of reactions run for 50 s, considering ε_{652}(CTC) = 39,000 $M^{-1}cm^{-1}$.[S5] For v_{in}(CTC) without added L-His, see **Figure S27**.

14. References

(S1) Corrin, M. L.; Harkins, W. D. Determination of the Critical Concentration for Micelle Formation in Solutions of Colloidal Electrolytes by the Spectral Change of a Dye. *J. Am. Chem. Soc.* **1947**, *69*, 679–683.

(S2) Takeda, K.; Tatsumoto, N.; Yasunaga, T. Kinetic Study on Solubilization of Pinacyanol Chloride into the Micelle of Sodium Dodecyl Sulfate by a Stopped-Flow Technique. *J. Colloid Interface Sci.* **1974**, *47*, 128–133.

(S3) Martínez de la Ossa, E.; Marchante, A. An Analysis of the Colorimetric Method of Determining CMC. *J. Dispersion Sci. Technol.* **1986**, *7*, 409−418.

(S4) Sabaté, R.; Estelrich, J. Determination of Micellar Microenvironment of Pinacyanol by Visible Spectroscopy. *J. Phys. Chem. B* **2003**, *107*, 4137−4142.

(S5) Josephy, P. D.; Eling, T.; Mason, R. P. The Horseradish Peroxidase-catalyzed Oxidation of 3,5,3',5'-Tetramethylbenzidine. Free Radical and Charge-transfer Complex Intermediates. *J. Biol. Chem.* **1982**, *257*, 3669–3675.

(S6) de Villiers, K. A.; Kaschula, C. H.; Egan, T. J.; Marques, H. M. Speciation and structure of ferriprotoporphyrin IX in aqueous solution: spectroscopic and diffusion measurements demonstrate dimerization, but not μ-oxo dimer formation. *JBIC, J. Biol. Inorg. Chem.* **2007**, *12*, 101–117.

(S7) Ma, J.-G.; Boyd, B. J.; Drummond, C. J. Positional Isomers of Linear Sodium Dodecyl Benzene Sulfonate: Solubility, Self-Assembly, and Air/Water Interfacial Activity. *Langmuir* **2006**, *22*, 8646−8654.

(S8) Guo, Z.; Rüegger, H.; Kissner, R.; Ishikawa, T.; Willeke, M.; Walde, P. Vesicles as Soft Templates for the Enzymatic Polymerization of Aniline. *Langmuir* **2009**, *25*, 11390−11405.

(S9) Ghéczy, N.; Sasaki, K.; Yoshimoto, M.; Pour-Esmaeil, S.; Kröger, M.; Stano, P.; Walde, P. A two-enzyme cascade reaction consisting of two reaction pathways. Studies in bulk solution for understanding the performance of a flow-through device with immobilised enzymes. *RSC Adv.* **2020**, *10*, 18655−18676.

(S10) Childs, R. E.; Bardsley, W. G. The Steady-State Kinetics of Peroxidase with 2,2'-Azino-di-(3-ethylbenzthiazoline-6-sulphonic acid) as Chromogen. *Biochem. J.* **1975**, *145*, 93−103.

(S11) Cvjetan, N.; Walde, P. Ferric heme *b* in aqueous micellar and vesicular systems: state-of-the-art and challenges. *Q. Rev. Biophys.* **2023**, *56*, e1, 1−43.

(S12) Oja, S. M.; Guerrette, J. P.; David, M. R.; Zhang, B. Fluorescence-Enabled Electrochemical Microscopy with Dihydroresorufin as a Fluorogenic Indicator. *Anal. Chem.* **2014**, *86*, 6040−6048.

(S13) Hargreaves, W. R.; Deamer, D. W. Liposomes from Ionic, Single-Chain Amphiphiles. *Biochemistry* **1978**, *17*, 3759−3768.

(S14) MacDonald, R. C.; MacDonald, R. I.; Menco, B. Ph. M.; Takeshita, K.; Subbarao, N. K.; Hu, L.-R. Small-volume extrusion apparatus for preparation of large, unilamellar vesicles. *Biochim. Biophys. Acta* **1991**, *1061*, 297−303.

15. Contents of the Supporting Movies

Movie S1

MD simulations (150 ns) indicating persistence of the interactions between hemin and SDS and between hemin and HEPES in water at pH = 7.2. Only the three entities − hemin, SDS, and HEPES − are made visible, with hemin kept in central place by fixing the position of three atoms of the porphyrin ring on the right-hand side.

Movie S2

MD simulation of a box (3,000 nm^3) containing 180 HEPES molecules (100 M) with 60 Na^+ counter ions, 70 SDS molecules (35 M) with 70 Na+ counter ions, 1 hemin with 1 Cl^- and 2 Na^+ counter ions, 21 DMSO molecules (11M), and 31,692 water molecules. The porphyrin ring of hemin is shown in magenta. Please note that for obvious reasons, the simulation box must contain at least one hemin to show possible interactions between hemin and SDS and hemin and HEPES. One hemin randomly placed inside the simulation box corresponds to a concentration of 533 mM, which is much higher than the concentration of hemin used in the (wet) experiments. In addition, the HEPES, SDS, and DMSO concentrations were also higher than in the experiments, see also **Figure S2**.
